# Nature inspired design methodology for a wide field of view achromatic metalens

**DOI:** 10.1515/nanoph-2025-0279

**Published:** 2025-10-13

**Authors:** Jacob Engelberg, Ronen Mazurski, Uriel Levy

**Affiliations:** Department of Applied Physics, The Faculty of Science, 98519 The Hebrew University of Jerusalem, 9190401, Jerusalem, Israel; Department of Electro-Optics and Applied Physics, Jerusalem College of Technology, 9116001, Jerusalem, Israel

**Keywords:** metalens, achromatic, wide-field-of-view

## Abstract

Metalenses have become ubiquitous in academic research and have begun to make their transition to industry. However, chromatic aberration still greatly limits the applications of metalenses. Achieving a wide field-of-view (FOV) is another challenge that has been dealt with successfully by using a removed stop, but when combined with a broadband spectrum, lateral chromatic aberration severely limits performance. In this paper, we tackle this challenge and present a comprehensive design methodology for a simultaneously wide-FOV and achromatic metalens, which is inspired by the human visual system. As a design example, we present a metalens operating in the near infrared (NIR), with 10 % relative spectral bandwidth (807–893 nm), focal length of 5 mm, F/5, and FOV of ±20°. In particular, we show how to optimize the stop position and correct the lateral chromatic aberration, both of which have not been reported in the past. In addition, we evaluate the performance of the metalens using accurate performance metrics and demonstrate the improvement compared to a chromatic metalens. Our approach paves the way for the design of wide FOV metalenses that can operate over a relatively large bandwidth, effectively contributing to the widespread implementation of metalens science and technology.

## 1 Introduction

Metalenses have been attracting a lot of attention over the last decade, when they were implemented using dielectric materials thus obtaining high efficiency [1], [2]. It was quickly realized that since metalenses are a type of diffractive lens (i.e., their phase function is not continuous but rather modulo a multiple of 2π), they are severely limited by chromatic aberration [3]. Thus, the race for implementing achromatic metalenses began. Several methods were originally proposed: spatial multiplexing [4], [5], cascading [6], and dispersion engineering, where the latter quickly became the dominant method used [7], [8], [9], [10]. At first it seemed that the problem was solved, but it was soon shown that dispersion engineering was limited to small metalens apertures [11], or more accurately, to low Fresnel numbers [12], [13].

In addition to the work on correcting metalens axial chromatic aberration, a parallel path was being pursued to achieve wide FOV metalenses, necessary for most imaging applications. This entails correcting the monochromatic off-axis aberrations. A solution was found in [14], [15] based on [16], where it was shown that a removed aperture stop located at the front focal plane of the metalens allows for correction of the third-order off-axis aberrations (coma and astigmatism). Of course, there is a price paid here: two optical elements, spaced apart, are needed, rather than one (in the case of [14], there is another metalens located at the stop, but even the stop itself, as in [15], can be considered as another element). Thus, the metalens system is no longer so thin. On the other hand, even without the additional element, there is still the distance between the metalens and the sensor, so the overall system length has only been approximately doubled. There have been attempts to remove this price-tag, by applying a synthetic removed aperture using total internal reflection [17] or wavevector filter [18]. The drawback of the first method is that it has large spherical aberration, which degrades resolution severely. While this could be corrected with image processing using deconvolution, in a real scenario image quality would be limited by noise. The second method seems to work nicely but is limited to monochromatic applications.

**Figure 1:**
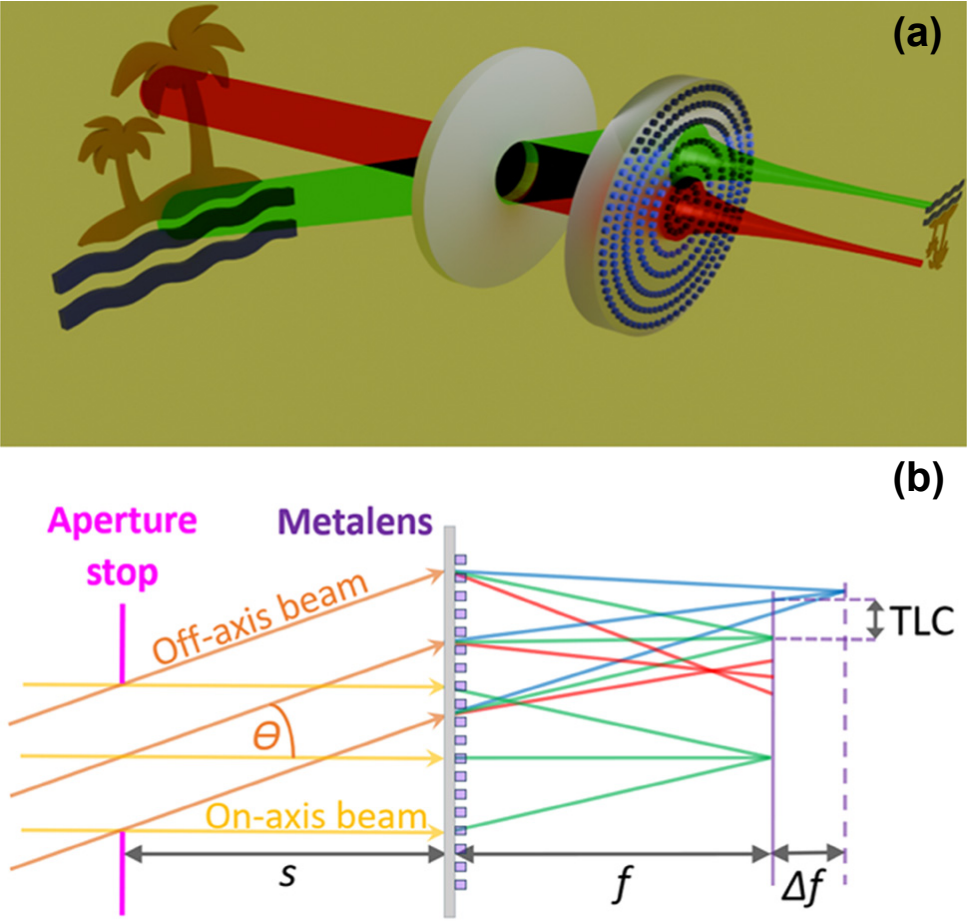
Wide-FOV metalens concept. (a) Sketch of a landscape type metalens system. (b) Drawing showing off-axis chromatic aberrations at the center wavelength focal plane, located at a distance f to the right of the lens, for the case of a removed aperture stop located at distance s to the left of the lens. Both axial (defocus Δf of blue and red rays) and lateral (displacement of chief ray, TLC, along the radial direction) chromatic aberrations affect the off-axis beam.

The design of a metalens, which is both wide-FOV and achromatic, is very challenging. While the correction of chromatic aberration is challenging even when restricting its operation to on-axis applications, it becomes even more difficult off-axis. The reason for this is that the removed stop solution for the monochromatic off-axis aberrations introduces a new off-axis aberration: Lateral chromatic, i.e., the focal spot experiences a wavelength dependent lateral shift (on top of the axial shift, see Figure 1b). In principle, this aberration could be corrected using dispersion engineered nanostructures, as was used to correct the on-axis chromatic aberration. Yet, for a wide-FOV metalens with a removed stop, there is pupil wander at the metalens surface, increasing the size of the metalens (Figure 1). This, in turn, increases the effective Fresnel number of the metalens, making it more difficult to correct the chromatic aberration [13].

An important issue in the design of metalenses is the method used to evaluate their performance. The two most important performance parameters are resolution and signal-to-noise ratio (SNR). In [19], we pointed out that the commonly used resolution metric of full-width half-maximum (FWHM) of the point-spread-function (PSF) is not meaningful in most cases, because the aberrations tend to move energy into the PSF sidelobes, without significant change to the FWHM. The commonly used “focusing efficiency” metric, which represents the signal part of the SNR, tends to mix resolution and signal level in an undetermined manner, because it clips the power in the PSF at a certain image radius (usually defined as 3·FWHM), regardless of the actual extent of the PSF. It also lacks any regard for background illumination, which contributes to the noise level (i.e., the “focusing efficiency” metric will not differentiate between light reflected/absorbed by the metalens and light transmitted to other diffraction orders, the latter contributing to background illumination and, therefore, to noise).

The performance of the achromatic metalens presented in this manuscript will be evaluated based on the metrics introduced in [20]. These metrics include Strehl ratio as a measure of resolution, diffraction efficiency (*η*) as a measure of signal level, and overall transmission (*T*) as a measure of noise level. However, when evaluating achromatic metalenses, this is still not enough, since usually they improve resolution by correcting the chromatic aberration at the expense of efficiency, which is reduced because the ideal blaze contour cannot be followed accurately at all wavelengths. This motivated us to develop an overall-performance-metric (OPM) that combines these three metrics into a single performance metric, proportional to the SNR averaged over all spatial frequencies, according to Eq. (1) [19], [20]. OPM values are between 0 and 1. A value of 1 represents an ideal situation where the Strehl ratio, transmission, and efficiency are all 1.

(1) $$\text{OPM}=\frac{\eta }{\sqrt{T}}\cdot \text{Strehl}$$

There have recently been some reports of wide-FOV achromatic metalenses, using a removed stop and dispersion engineering [21], [22], [23], [24]. In this paper, we describe an achromatic wide-FOV metalens for the near-infrared (NIR) spectral range, focal length 5 mm, F-number 5 (F/5), and total FOV 40°. The main novelty of this paper with respect to previous reports is that we account for lateral chromatic aberration and optimize the stop location. Additional contributions of this paper are as follows: (a) We design the lens with large dimensions, appropriate for real-world applications, such as security cameras. (b) We evaluate the lens using real-world performance measures that account not only for resolution but also for signal-to-noise ratio (SNR) [20]. (c) We introduced a phase jump along the metalens aperture and found that we were able to significantly improve the center-FOV performance using this method. This approach is inspired by nature, e.g., the human visual system, which provides high resolution in the center of the FOV (fovea region), at the expense of lower resolution in the peripheral region, which is mostly used for tasks such as orientation and motion detection [25]. The limiting resolution of the human eye as a function of field and modulation transfer-functions (MTF) for different FOVs is shown in Figures S1-S2.

## 2 Macro-level optical system design

We designed a relatively large scale, 5 mm effective focal length (EFL), F/5, 40° full-FOV, achromatic metalens. We chose a wavelength range centered on 850 nm with a spectral width of 86 nm (∼10 % relative spectral width), which can be relevant for day or night-time surveillance (the former with an appropriate band-pass filter and the latter with LED illumination). The most successful method proposed to date to correct monochromatic off-axis aberrations and obtain a wide-FOV using flat lenses is the landscape telecentric lens originally proposed by Buralli and Morris [16]. This method was implemented for a metalens doublet in [14] and for a single metalens in [15]. The method consists of an iris located at the front focal plane of the metalens, as shown schematically in Figure 1. Note the pupil wander, i.e., the location of the off-axis beam on the lens aperture shifts away from the axis, increasing the required metalens diameter.

The landscape lens method has two main drawbacks: (a) The larger the FOV, the larger the pupil wander on the metalens surface, requiring a large metalens aperture. (b) Lateral chromatic aberration, proportional to the stop distance *s*, is introduced off-axis as shown in Figure 1b [3], [15]. These drawbacks can be mitigated if we can place the stop nearer to the metalens, with the additional advantage of decreasing overall system length. The second drawback can be additionally diminished if we are able to somehow achromatize the metalens. The second drawback indicates that the optimal stop location for a broadband metalens may be closer to the metalens than for a monochromatic metalens, since there is a tradeoff between the monochromatic and chromatic aberrations as we shift the stop position [3].

**Figure 2:**
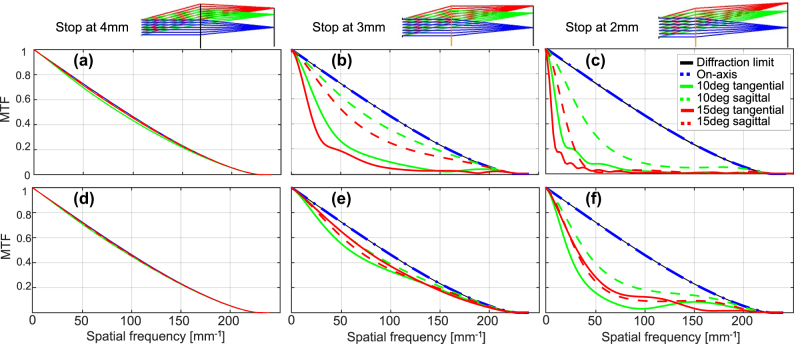
Monochromatic MTF at different stop distances for our 5 mm EFL F/5 metalens – comparison of quadratic to high-order phase function. (a–c) MTF for a quadratic phase, at stop distances of 4, 3, and 2 mm from the metalens, respectively. (d–f) MTF for optimized high-order phase, for the same stop distances. The insets at the top show the design layout for the different stop distances.

Buralli and Morris referred to the case of a monochromatic metalens with quadratic phase ( $\phi \left(r\right)=Ar^{2}$ ) and concluded that the optimal stop position is at the front focal plane of the diffractive lens, resulting in perfect correction of all the off-axis third order aberrations (except distortion, but this aberration does not affect resolution) [16]. However, by using higher order phase coefficients ( $\phi \left(r\right)=A_{1}r^{2}+A_{2}r^{4}+\cdots \;$ ), the correction of the monochromatic aberrations at closer stop distances can be significantly improved. We demonstrate this capability in Figure 2, which compares the MTF for a quadratic versus high-order polynomial phase metalens. The design and simulation were done for monochromatic illumination (850 nm), using Zemax optical design software. For the optimization, we used the default Zemax wavefront merit function with equally weighted field angles of 0°, 5°, 10°, 15°, and 20°.

For a monochromatic metalens, the optimal stop position from an optical performance point-of-view is clearly still at the front focal plane. However, for the sake of compactness, it may well be desired to use the design of Figure 2e, i.e., a stop distance of 3 mm. As can be seen, the degradation of optical performance is negligible, whereas the overall length of the optical system, from stop to image, can be reduced by about 20 %, from ∼10 mm to ∼8 mm. Note that in our design, we neglected the substrate thickness, taking it as zero. Neglecting the substrate thickness allowed us to better demonstrate the dependence of performance on stop position, without the extra complication of optical thickness of the substrate. In a real metalens, we will of course need to introduce a mechanically feasible thickness. To demonstrate this, in Figures S3 and S4 of the Supplementary, we present the optical prescription of the design shown in Figure 2e, and an equivalent design with a 1 mm sapphire substrate and a stop 2.5 mm away from the substrate front surface. This stop-location corresponds closely to an “optical” distance of 3 mm from the iris to the metalens, equivalent to the substrate-less design of Figure 2e, as shown in Section 2 of the Supplementary. The method of design and optimization used for the “real” system with a substrate is identical to that of the ideal substrate-less system.

**Figure 3:**
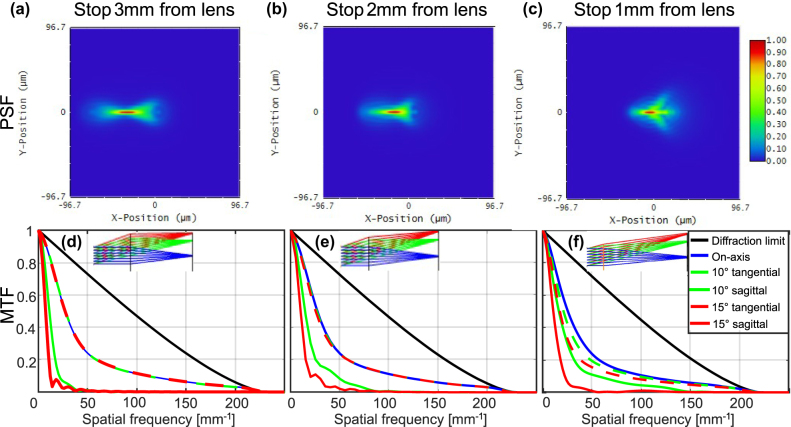
Polychromatic (810–890 nm) PSFs and MTFs for different stop locations. (a, d) PSF at 15° field point and MTFs for stop distance of 3 mm, respectively. (b, e) PSF at 15° field point and MTFs for stop distance of 2 mm, respectively. (c, f) PSF at 15° field point and MTFs for stop distance of 1 mm, respectively. The optimum Strehl ratio is obtained at stop distance of 2 mm. The insets show the design layout for the different stop distances.

So far, we have discussed monochromatic operation of our metalens. For a polychromatic metalens, the optimal stop position will likely be obtained at a shorter stop distance, as decrease in stop distance allows reduction of the lateral chromatic aberration. To demonstrate this, we first look at the extreme case of a “chromatic” metalens, meaning a metalens with no chromatic correction. In this case, the optimal stop position will be closest to the metalens, to reduce the lateral chromatic aberration. Zemax simulation results for the designs of Figure 2e and f, and an additional design for 1 mm stop distance is shown in Figure 3. The simulations use 21 equally spaced and equally weighted wavelengths in the range 810–890 nm. It turns out that for a “chromatic” implementation of this metalens, the optimal stop position (based on the Strehl ratio at 15° FOV) shifts from 3 mm away from the lens to only 2 mm. While this is difficult to discern visually from the PSFs and MTFs shown in Figure 3, it can be easily extracted from the numerical Strehl ratio values in Figure 5b. Note that while the variation in performance around the optimum stop location is fairly small, one can still observe (Figure 3a–c) that the performance goes from being limited by lateral chromatic at 3 mm stop distance (more horizontally elongated PSF) to being limited by coma at 1 mm distance (more comatic shape).

## 3 Nanostructure dispersion-engineered design

Following the macro-level metalens design, we now discuss the nano-level design, where we perform chromatic correction using dispersion engineering [8], [9], [11]. Normally, when light propagates a distance *H* through a truncated waveguide type nanostructure, the phase dispersion (d*ø*/d*ω*) is positive, because $\phi \left(\omega \right)=\frac{\omega }{c}n_{\text{eff}}\left(\omega \right)H$ and d*n*_eff_/d*ω* is positive (the larger the frequency the higher the mode confinement, so the higher the effective refractive index, *n*_eff_) [13]. This type of dispersion is useful for correcting the chromatic aberration of a metalens, since the higher frequencies (shorter wavelengths) tend to focus less (weaker diffraction), so we need to give them more phase. However, as was shown by Shrestha et al. [11], if the zero-phase point is defined at the center of the lens, the phase induced by a positive (focusing) metalens needs to be negative. This means that to correct chromatic aberration, we need the phase to be more negative (larger absolute value) for higher frequencies, i.e., we require *negative* phase dispersion. Since such structures are not available, it was suggested by Shrestha to overcome this by adding a frequency-dependent phase bias, C(*ω*), which shifts the zero phase to the edge of the lens. Now the required dispersion is positive, which can be accommodated by typical nanostructures. We used this method, of applying different phase pistons for the different wavelengths, so that the phase for all wavelengths is identical at the edge of the aperture.

While the above achromatization method can work well for small scale lenses, when we try to design a large diameter metalens, the rapidly changing phase for each wavelength as we move away from the optical axis necessitates high-dispersion nanostructures near the center of the lens so that each wavelength can follow its phase function. If the required dispersion exceeds the theoretical limits [12], or those of the specific nanostructure library, the metalens performance will degrade. Therefore, as the diameter of the metalens increases, it becomes more difficult to obtain good polychromatic performance.

As can be seen in Figure 1, despite our modest F-number of 5, the FOV requirement increases the metalens diameter because of pupil wander, making it difficult to achieve good achromatic performance. We found that when we increase the diameter of the metalens to obtain large FOV, we damage the on-axis imaging, as it becomes harder to fit the correct phase for all the wavelengths simultaneously. However, we can leverage the fact that each field-point only uses part of the aperture, to introduce a phase jump at a certain aperture radius. The phase jump is implemented by changing the phase bias *C*(*ω*) at this aperture radius. We selected the jump location to be at the edge of the on-axis beam, thus obtaining optimal performance on-axis, which mimics the human vision system. More detailed information about our nanoscale design can be found in the Materials and Methods section.

## 4 Results

The performance of our achromatic metalens was evaluated at 33 wavelengths within our spectral range (807–893 nm), and their results were averaged to obtain the polychromatic performance. The PSF for each wavelength was calculated using angular spectrum propagation [26] from the metalens to the image plane, and the PSFs were summed to obtain the polychromatic PSF. The pupil phase function after the metalens for each field point was calculated based on the geometrical path length of the rays accounting for the wavefront tilt of each field point (multiplied by 2*π*/*λ* to convert to the path length to phase for each wavelength *λ*), and adding the metalens phase at that wavelength, for each radial position on the metalens, as described by Eq. (3) in the Materials and Methods section.

The overall transmission *T* is calculated based on the spectral transmission of each of the nanostructures calculated in Lumerical Finite-Difference-Time-Domain (FDTD) software. The diffraction efficiency *η* is the fraction of light arriving at the first order of diffraction based on the angular propagation simulation result, relative to the incident light. To calculate it, we first calculated the “focusing efficiency,” which we define as the fraction of light arriving at the first order of diffraction relative to the light transmitted through the metalens. Denoting the focusing efficiency as *η*_*f*_, one obtains: *η* = *η*_*f*_ *T*. The focusing efficiency is calculated as the sum of the PSF values, within a small region in the image plane, until the PSF intensity flattens out [19], divided by the sum of the entire simulation region on the image plane (a square with side-length of 4 mm, which is 4 times the aperture diameter, so it includes all the zero and second order light, and much of the other orders). Note that this definition of focusing efficiency is different from that commonly used by other authors, which represents an unknown mixture of resolution and efficiency [19]. The Strehl ratio used here is the volume under the 2-dimensional (2D) MTF relative to the volume under the diffraction limited MTF (the official definition is based on PSF peak, but the MTF-based definition is equivalent for the on-axis case, and suitable for our purposes also in the off-axis case [20]).

**Figure 4:**
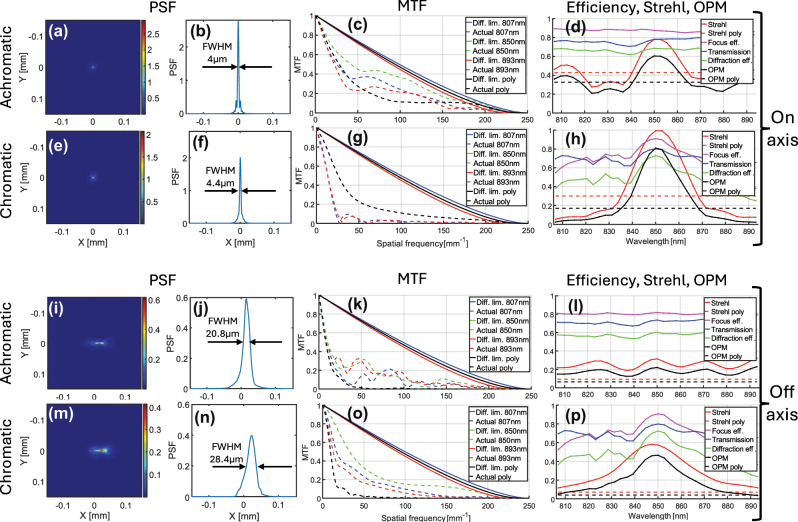
On-axis (a–h) and 15° off-axis (i–p) performance comparison between achromatic (a–d, i–l) and chromatic (e–h, m–p) metalenses. (a, e, I, m) 2D PSFs normalized so that each volume under the function is equal to 1, and (b, f, j, n) their cross sections, respectively. FWHM is marked on each PSF’s cross section. (c, g, k, o) MTFs comparison, solid lines represent diffraction limited MTFs, and dashed lines are the achieved MTFs. The dashed black lines are the polychromatic MTFs. (d, h, l, p) Strehl, efficiency, and overall performance (OPM) comparison as a function of wavelength. Evaluation is at the optimal stop distance, 2 mm for the chromatic and 2.5 mm for the achromatic metalens.

The on-axis PSF of our achromatic metalens is shown in Figure 4a and b, compared to the PSF of the equivalent chromatic metalens in Figure 4e and f. The achromatic metalens shows a similar full-width at half-maximum (FWHM) value, but reduced energy in the sidelobes, which corresponds to the higher PSF peak and, therefore, higher polychromatic Strehl ratio, as shown in Figure 4d and h. This is a result of correction of the axial chromatic aberration. All PSFs shown in the main manuscript are polychromatic. PSFs for three discrete wavelengths (center and extreme) are shown in Figure S13.

Figure 4c and g compares the metalens MTFs on-axis. MTFs at three specific wavelengths (center and extreme wavelengths) are shown. In addition, the polychromatic MTF, calculated from the polychromatic PSF, averaged over 33 wavelengths within the range with uniform weighting, is shown. The chromatic metalens (panel (g)) has diffraction-limited (DL) MTF at the central wavelength (850 nm), but poor MTF at the extreme wavelengths because of chromatic aberration, resulting in poor polychromatic MTF. In contrast, while the achromatic metalens MTFs are below DL, they are similar for all wavelengths (panel (c)), resulting in an improved polychromatic MTF (27.4 % versus 22.8 % at 50 c/mm, about the same at 100 c/mm, but again much improved at spatial frequencies above 100 c/mm).

Figure 4d and h compares the on-axis Strehl ratio, transmission, focusing efficiency, diffraction efficiency, and OPM as a function of wavelength for achromatic versus chromatic metalenses, respectively. In addition to the results for each wavelength, we also show the polychromatic results for these metrics, which are represented as a flat line, as they are not wavelength dependent but are rather the average over all the wavelengths. Here too, the chromatic metalens achieves excellent results at the nominal wavelength but degrades quickly as the wavelength departs from the nominal, so that the polychromatic performance is poor. In contrast, our achromatic metalens gives more uniform performance over all wavelengths, achieving a polychromatic OPM of 0.33. This is approximately a twofold improvement over the OPM value of 0.17 for the chromatic metalens.

Off-axis, the performance is dependent on the stop distance from the lens, as shown in Figure 3 for the case of a chromatic lens. The optimal stop position is the one where the lateral chromatic aberration (which is smaller for short stop distance) and the monochromatic off-axis aberrations (which are smaller for long stop distance) are balanced. For an achromatic metalens, we can expect the optimal stop distance to be farther from the metalens than for the chromatic metalens, since the lateral chromatic aberration is reduced.

**Figure 5:**
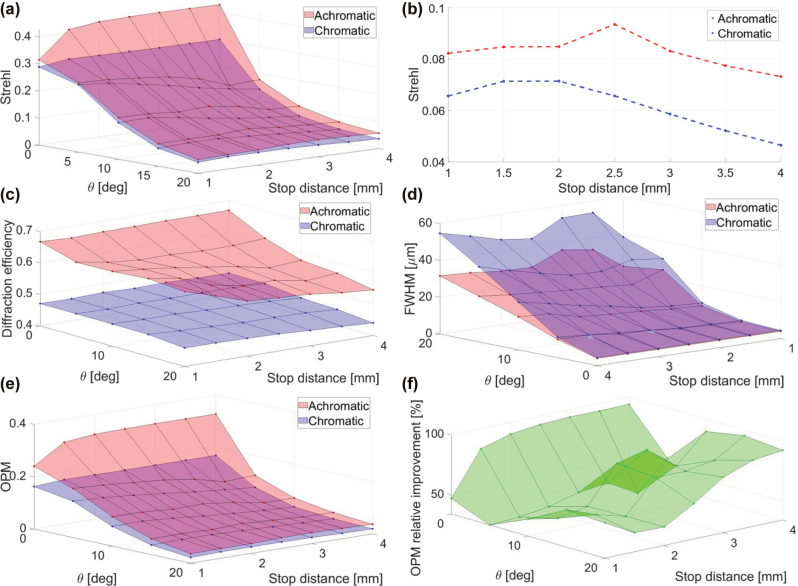
Performance comparison between achromatic and chromatic metalenses as a function of field angle (θ) and stop distance. (a–b) Strehl ratio full raw data and cross section at 15°, respectively. (c) Diffraction efficiency (*η*). (d) FWHM in the radial direction (note that the axes in this graph are reversed compared to the others). (e–f) OPM full raw data and relative improvement, respectively.

Figure 4i–p shows results for off-axis performance, at 15° field angle, using the same performance parameters as for the on-axis case. The results shown are for the optimal stop position of both metalenses, which turn out to be 2.5 mm for the achromatic metalens and 2 mm for the chromatic metalens – see Figure 5b. For the off-axis case, the average Strehl/OPM is significantly worse than would be expected by looking at the average of the values for each wavelength. This is because the lateral chromatic aberration introduces a lateral shift between the PSFs of the different wavelengths, meaning that despite good Strehl per wavelength, their spots don’t overlap, so their sum gives a large blur in the radial direction. The MTFs shown are only in the radial (tangential) direction, but the Strehl ratio is calculated based on the 2D MTF, so the Strehl and the OPM are accurate. Polychromatic performance, for other field angles of 5°, 10°, and 20°, is shown in Figures S7-S12, respectively. Off-axis performance at single wavelengths, for field angles of 5°, 10°, 15°, and 20°, is shown in Figures S14-S17, respectively.

An interesting point to note is that despite the modest improvement in polychromatic Strehl ratio, there is a significant change in the shape of the PSF cross section in the radial direction. It transforms from a more rounded peak in the chromatic case to a sharp peak in the achromatic case. This leads to a significant decrease in the FWHM of the achromatic metalens PSF cross section compared to that of the chromatic metalens as shown in Figure 4j and n. While in [19], [20] it was explained that in general the Strehl ratio is a better resolution performance metric than FWHM, there may be applications where the FWHM metric is important (for example, if we can threshold the image, ignoring whatever is below a given intensity, as is often done in imaging-based lithography systems).

Figure 5 compares the performance of the achromatic versus the chromatic metalens in terms of OPM, diffraction efficiency, FWHM, and Strehl ratio, for various stop distances and field angles. Figure 5a shows that for all cases, the achromatic metalens gives better resolution than its chromatic counterpart, yet the improvement is moderate. Figure 5b shows the cross section of Figure 5a at FOV of 15°, which we chose as our reference field-point for determining optimal stop position.

As opposed to the moderate improvement in Strehl ratio, the improvement in diffraction efficiency, shown in Figure 5c, is dramatic. This is of crucial importance, in particular for low light applications, where every photon counts. Furthermore, while for small field angles, the improved Strehl of the achromatic metalens does not significantly impact the radial FWHM, shown in Figure 5d, and for large field angles, the relative improvement in FWHM is significantly larger because of the lateral chromatic aberration correction. As previously mentioned, this might play a major role for applications where thresholding is applied. Finally, the overall performance and its relative improvement are shown in Figure 5e and f, respectively, demonstrating a major improvement over the chromatic metalens.

## 5 Discussion

The achromatic metalens presented in this paper was designed with parameters relevant to real world applications, such as a security camera lens. The emphasis of the paper is less on the specific performance achieved, but rather on the design methodology (e.g., use of a phase jump, optimizing the stop position, use of polychromatic PSF that includes the effect of lateral chromatic aberration, emphasizing the on-axis resolution, comparison to chromatic baseline), use of good performance metrics, and understanding the tradeoffs.

While the importance of a removed stop has long been understood [3], [14], [15], [16], it was generally accepted that it should be located at the front focal plane of the metalens to cancel the monochromatic third-order aberrations of coma and astigmatism. While there have been some publications where it was located closer to the metalens [22], [23], [24], no explanation was offered as to why this location was chosen. Here, we explained the tradeoffs for the case of a narrowband metalens (compactness versus performance) and for a broadband metalens (lateral chromatic aberration versus monochromatic aberrations) and demonstrated an optimization method.

We now briefly discuss the effect of different metalens parameters on our design methodology. The optimal stop location is determined by balancing the monochromatic and lateral chromatic aberration. As the F# increases, the monochromatic aberrations decrease, while the lateral chromatic aberration is unaffected [27]. Thus, the optimal stop location will be closer to the metalens. As for increasing the relative spectral range, this increases the lateral chromatic aberration [3] while not affecting the monochromatic aberrations. Thus, the optimal stop location will also be closer to the metalens.

An additional advantage of using a smaller stop distance is decreased metalens diameter and thus improved manufacturability (shorter write time and larger minimal zone width) and tolerance sensitivity (because of the smaller diffraction angles for the off-axis field points). A disadvantage of shifting the stop from the front focal plane is loss of telecentricity in the image domain, causing reduced image plane illumination uniformity because of the cosine-fourth law [27] and sensor angular response. All these considerations must be weighed depending on the design requirements.

In summary, as a design guideline, we suggest sweeping the stop distance from a short distance (which is desired from compactness and manufacturing considerations, and at which the lateral chromatic aberration will be minimized), toward larger distances (where monochromatic aberrations become more dominant), up to the point where the performance begins to degrade, and choose the distance that gives the optimum performance, considering the relevant parameters (Strehl ratio, metalens diameter, tolerances, illumination uniformity, etc.).

As explained in Section 3, we used a single phase jump in our metalens, at an aperture radius equal to that of the on-axis beam. Phase jumps have been implemented in some previously reported metalenses to compensate for the nanostructure phase dispersion, compared to required phase dispersion. In [28], this was done for the case of a narrow-FOV metalens operating at 3 discrete wavelengths. The phase jump location and magnitude (for each wavelength) were optimized to obtain optimum performance. In [24], phase jumps were used for a wide-FOV metalens doublet operating in the NIR, with phase jump locations determined by folding the ideal phase dispersion onto the available phase dispersion of the nanostructure library. In contrast, we use a nature inspired approach, providing optimal resolution in the center of the FOV by using a single-phase jump. By sacrificing resolution in the peripheral FOV, we gain high resolution in the center of the FOV, and much higher efficiency over the entire FOV (about 60 % in our design as compared to about 15 % in previous designs). To benefit from this approach, our metalens based surveillance system would have to be mounted on a motorized pan and tilt mechanism, as is common in commercial security cameras. Video motion detection can then be used to automatically rotate the system’s optical-axis so that the moving object will be at the center of the FOV.

Our results demonstrate that for most real-word applications, which require a focal length of at least a few millimeters and a significant FOV, chromatic aberration is still a severely limiting problem. This is despite the plethora of reports on achromatic metalenses. It would be interesting to compare our performance to that of other published broadband wide-FOV metalenses. In general, it is difficult to compare between different published designs since often proper performance metrics are not used, as discussed in Section 1 [19]. In addition, each publication has different parameters, so comparing the OPM directly would not be useful. However, in a previous publication, we introduced an extended OPM metric (EOPM) that accounts for the scaling of the metalens (via the focal length) and the relative spectral range [20], although still limited to on-axis performance. The EOPM for our achromatic metalens design is 7.1, higher than any of the achromatic metalenses analyzed in [20].

The design methodology used in this paper is a linear flow composed of two stages: macro-level optical design in Zemax followed by nano-level design using Lumerical and Matlab. In the nano-level design, we attempt to fit the nanostructure phase (for all wavelengths) to the phase determined in the macro-level Zemax design, with adjustments per Eq. (3). We expect it will be possible to further improve the design by adding an iterative stage where we directly optimize the nanostructures used at every radial position in Matlab, using our OPM metric as the merit function. We have experimented with this in an MWIR achromatic metalens design we are currently working on, and have obtained positive initial results, which we hope to report on in the future.

## 6 Materials and methods

**Figure 6:**
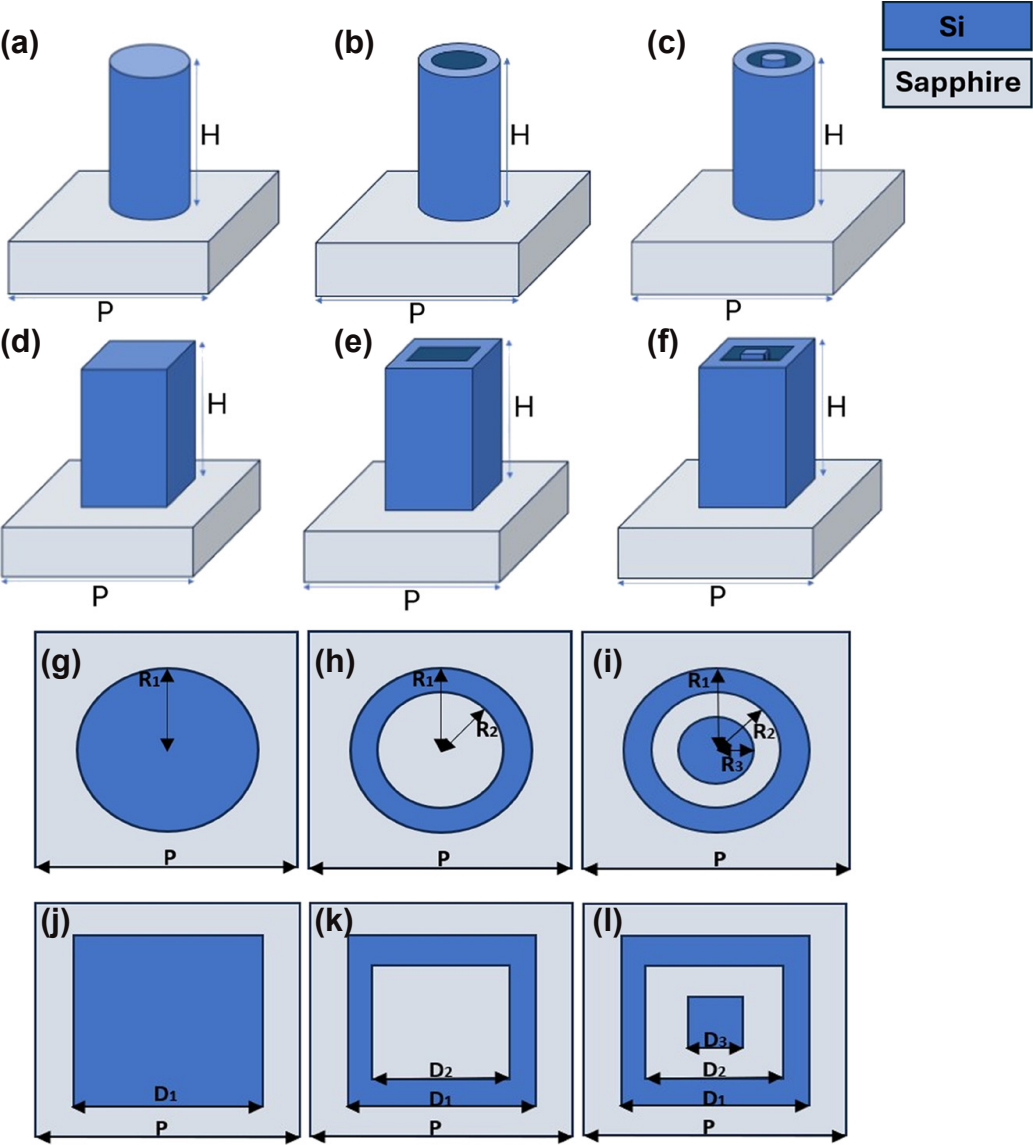
Nanostructure library for achromatic metalens. (a–f) Perspective views of the nanostructures. (g–l) Top views of the same nanostructures.

This section gives details of our nanostructure library design. To correct the chromatic aberrations of our metalens, we designed and simulated a library of periodic silicon nanostructures on a sapphire substrate using Lumerical Finite-Difference-Time-Domain (FDTD) software. The nanostructures were designed as truncated waveguides with varying shapes, as shown in Figure 6. The period (P) and the height (H) are constant for all pillars and are set to 0.4 µm and 1.5 µm, respectively. For the circular pillars, the radii dimensions were swept in the ranges: 0 < R_3_ < 160 nm, R_3_ < R_2_ < 180 nm, R_2_ < R_1_ < 200 nm. For the square pillars, the widths were swept in the ranges: 0 < D_3_ < 320 nm, D_3_ < D_2_ < 360 nm, D_2_ < D_1_ < 400 nm. The dimensions R_i_, D_i_ are as shown in Figure 6g–l.

**Figure 7:**
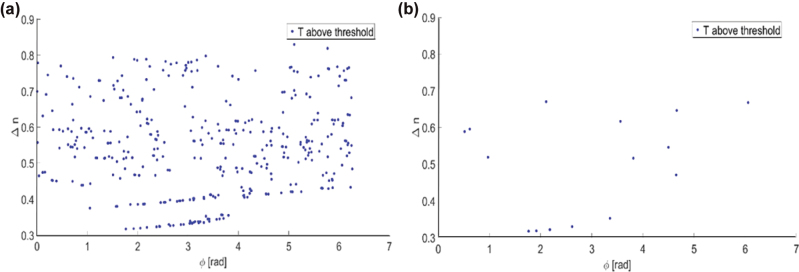
Dispersion in terms of Δn as a function of ϕ for (a) achromatic nanostructure library and (b) chromatic nanostructure library.

To evaluate our nanostructure library, we created the graph presented in Figure 7a, which shows the dispersion, Δn, as a function of the phase, φ, of the center wavelength. The dispersion is represented by the difference in effective refractive index between the extreme wavelengths. Each circle on the graph represents a specific nanostructure geometry. We display and use only nanostructures that provide transmission of above 40 %. In the following, we compare our achromatic metalens design to that of an equivalent chromatic metalens. For the chromatic metalens, we used simple cylindrical pillars, as presented in tiles (a) and (g) of Figure 6. Figure 7b shows the sparse dispersion graph for this library (a single dispersion value for each phase value). The nanostructure phases at the various wavelengths were evaluated using Lumerical FDTD software, at normal incidence only, under the assumption that the phase does not change much for other incidence angles. This allowed us to simplify the analysis and is reasonable, since it was shown in [29] that at moderate incidence angles (up to 20°) and NA (up to 0.13), truncated waveguide type metalens performance is maintained, indicating that the phase is close to that of normal incidence.

The achromatic metalens bandwidth limit formulated in [12] was translated to a limit on the maximum attainable Fresnel number (FN) of a diffraction limited achromatic metalens in [13] and is expressed in Eq. (2):

(2) $$\text{F}{\text{N}}_{\max}=2h\Delta n{\left(\frac{\Delta \lambda }{\lambda_{0}}\right)}^{-1}$$

Where *h* is the pillar height relative to the center wavelength, *λ*_*0*_, *Δn* is the maximum dispersion, and *Δλ/λ*_*0*_ is the relative spectral range. For our lens parameters, we have $h=\frac{H}{\lambda_{0}}=\frac{1,500}{850}=1.76$ , and *Δλ/λ*_*0*_ = 0.1. For *Δn*, the theoretically obtainable dispersions are in the range of 0–2.1. This is because our nanostructures are made of silicon (refractive index @850 nm ∼3.6), and in our design, we used a cover of PMMA (refractive index @850 nm ∼1.5). However, in practice the dispersion will always be lower because of fabrication constraints, for example, the nanostructure radii and the difference between them cannot be too small. Therefore, as can be seen in Figure 7a, we obtain a maximum dispersion of 0.83 and a dispersion range of 0.51 (since the minimum dispersion is not zero, but 0.32). Based on the above formula, and using Δ*n* = 0.51, the maximum FN comes out to be 18.

Let us compare this to the FN of our lens, which is given by [26]: $\text{FN}=\frac{R^{2}}{\lambda_{0}f}$ , where *R* is the lens aperture radius. In our case, we have $R=\frac{D}{2}=\frac{f}{2F}=\frac{5}{2.5}=0.5\;\text{mm}$ , and $\text{FN}=\frac{0.5^{2}}{0.00085.5}=59$ . To make matters worse, this is the case when we use the aperture stop radius to calculate the FN. However, as we need to accommodate the pupil wander off-axis, we obtain a much larger FN, whose magnitude depends on the stop distance. It is clear, therefore, that we cannot hope to obtain diffraction limited performance using these lens parameters. However, we can hope to make a significant improvement in performance compared to a chromatic metalens.

To evaluate the improvement our achromatic metalens provides over a standard chromatic metalens, we designed a competing chromatic metalens using cylindrical pillars. For the design of the chromatic metalens, we chose for each radial position the pillar radius that most accurately achieves the required phase at the nominal wavelength, with no regard for the phase at other wavelengths. For the design of the achromatic metalens, we searched the extended library for the nanostructure that most accurately achieves the required phase at 5 wavelengths, evenly spaced in the range 807–893 nm, with weights of [1, 1.2, 1.5, 1.2, 1].

**Figure 8:**
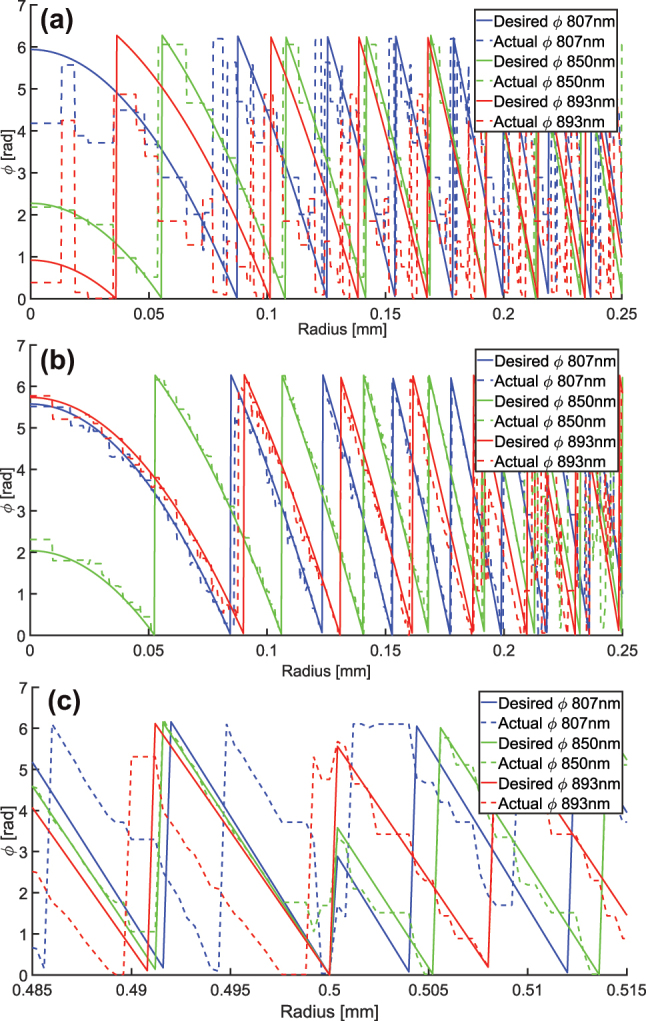
Phase fitting of the metalens for (a) chromatic metalens, and (b) achromatic metalens – both at the center of the aperture, and (c) achromatic metalens around 0.5 mm aperture radius. Solid lines represent the ideal desired phase for each wavelength, and dashed lines represent the phase that the chosen nanostructure achieves for each wavelength at each point along the aperture radius.

Figure 8 shows the actual phase profiles obtained for the achromatic and chromatic metalens, based on their respective nanostructure libraries, compared to the theoretically desired phase profiles, which are given by Eq. (3):

(3) $$\begin{array}{ll} \phi \left(r,\lambda \right) & =\frac{\lambda_{0}}{\lambda }\phi \left(r,\lambda_{0}\right)+C\left(\lambda \right)\\ & =\frac{\lambda_{0}}{\lambda }\left(A_{1}r^{2}+A_{2}r^{4}+\ldots \right)+C\left(\lambda \right) \end{array}$$

Where *λ*_0_ is the design wavelength (in our case 850 nm), *A*_*i*_ are the phase coefficients from Zemax, and $C\left(\lambda \right)$ is the previously mentioned phase bias. The *λ*_0_/*λ* factor comes from dividing the phase at the design wavelength, $\phi \left(r,\lambda_{0}\right)$ , by 2*π*/*λ*_0_ to convert the phase to optical path difference (OPD), which should be constant for all wavelengths in an achromatic lens, and then multiplying by 2*π*/*λ* to obtain the corresponding phase at each wavelength. As mentioned in Section 3, we applied a single phase jump at the edge of the on-axis aperture radius (0.5 mm in our case), meaning we use Eq. (3) with two different $C\left(\lambda \right)$ functions, one for aperture radius 0–0.5 mm, and another for aperture radius 0.5 to the edge of the lens (1.41 mm in our case).

Figure 8 panels (a) and (b) compare the phase of the chromatic and achromatic metalenses, respectively, in the central 0.25 mm of the aperture radius (at larger apertures the zones are very dense, so it is difficult to visualize the phase). It can be seen that near the center of the aperture, the expanded nanostructure library of the achromatic metalens allows us to follow the ideal phase much more accurately for all the wavelengths. This improves not only the resolution of the metalens by correcting chromatic aberration but also the efficiency, by better matching the zone profile to the ideal profile. Figure 8c shows the desired and actual phase at an aperture radius of 0.5 mm, which is the edge of the on-axis aperture and the location of our phase jump. The desired phase for all three wavelengths at this aperture radius is zero, since we used this constraint to ensure that positive nanopillar dispersion is required, as explained in Section 3. However, immediately after this aperture radius, the desired phase jumps to a different value for each wavelength. This value is derived from the constraint that the phase for all wavelengths be again zero at the edge of the full metalens aperture. Both these zero-phase constraints are not the only option that can be used, but we found that they work well.

The effect of the phase jump on the achromatic metalens performance is discussed in Section 3 of the Supplementary. In short, Table S1 and Figure S5 show that the phase jump yields significant improvement in on-axis performance, without loss in off-axis performance, compared to the equivalent design without a phase jump. We did not explore the effect of adding additional phase jumps, since our primary focus was on optimizing the on-axis performance, so this may be a subject for further study. However, it is our feeling that it will be difficult to achieve significant improvement in the off-axis performance, since the improved phase fitting will be countered by the performance degradation caused by the phase jumps (see Figure S6 and discussion in Supplementary).

## 7 Conclusions

In this paper, we presented a nature inspired design method and rigorous performance analysis of a dispersion engineered achromatic wide-FOV metalens for the NIR spectral range. As such, the paper provides a recipe for the design of an achromatic metalens, and for properly evaluating its performance. Based on this approach, we have designed a specific wide-FOV achromatic metalens with a fractional bandwidth of 10 % operating in the NIR spectral range. Like the human visual system, our design provides high resolution on axis, at the expense of lower resolution in the peripheral FOV. This allows a significant improvement in performance compared to both conventional chromatic metalenses and previous achromatic designs. In addition to the improved resolution, we also achieved high efficiency of about 60 % over the entire wavelength range. We hope that the insights from this paper, including the factors limiting the performance, and the use of rigorous methods of evaluation, will facilitate future advances in the field.

## Supplementary Material

Supplementary Material Details

## References

[1] Khorasaninejad M., Chen W. T., Devlin R. C., Oh J., Zhu A. Y., Capasso F. (2016). Metalenses at visible wavelengths: Diffraction-limited focusing and subwavelength resolution imaging. *Science*.

[2] Lalanne P., Chavel P. (2017). Metalenses at visible wavelengths: past, present, perspectives. *Laser Photon. Rev.*.

[3] Engelberg J., Levy U. (2017). Optimizing the spectral range of diffractive metalenses for polychromatic imaging applications. *Opt. Express*.

[4] Arbabi E., Arbabi A., Kamali S. M., Horie Y., Faraon A. (2016). Multiwavelength polarization-insensitive lenses based on dielectric metasurfaces with meta-molecules. *Optica*.

[5] Arbabi E., Arbabi A., Kamali S. M., Horie Y., Faraon A. (2016). Multiwavelength metasurfaces through spatial multiplexing. *Sci. Rep.*.

[6] Avayu O., Almeida E., Prior Y., Ellenbogen T. (2017). Composite functional metasurfaces for multispectral achromatic optics. *Nat. Commun.*.

[7] Khorasaninejad M. (2017). Achromatic metalens over 60 nm bandwidth in the visible and metalens with reverse chromatic dispersion. *Nano Lett.*.

[8] Arbabi E., Arbabi A., Kamali S. M., Horie Y., Faraon A. (2017). Controlling the sign of chromatic dispersion in diffractive optics with dielectric metasurfaces. *Optica*.

[9] Chen W. T. (2018). A broadband achromatic metalens for focusing and imaging in the visible. *Nat. Nanotechnol*..

[10] Wang S. (2018). A broadband achromatic metalens in the visible. *Nat. Nanotechnol*..

[11] Shrestha S., Overvig A. C., Lu M., Stein A., Yu N. (2018). Broadband achromatic dielectric metalenses. *Light Sci. Appl.*.

[12] Presutti F., Monticone F. (2020). Focusing on bandwidth: achromatic metalens limits. *Optica*.

[13] Engelberg J., Levy U. (2021). Achromatic flat lens performance limits. *Optica*.

[14] Arbabi A., Arbabi E., Kamali S. M., Horie Y., Han S., Faraon A. (2016). Miniature optical planar camera based on a wide-angle metasurface doublet corrected for monochromatic aberrations. *Nat. Commun.*.

[15] Engelberg J., Zhou C., Mazurski N., Bar-David J., Kristensen A., Levy U. (2020). Near-IR wide-field-of-view Huygens metalens for outdoor imaging applications. *Nanophotonics*.

[16] Buralli D. A., Morris G. M. (1989). Design of a wide field diffractive landscape lens. *Appl. Opt.*.

[17] Martins A. (2020). On metalenses with arbitrarily wide field of view. *ACS Photonics*.

[18] Xie T. (2023). Ultrathin wide-angle and high-resolution meta-imaging system via rear-position wavevector filter. *Laser Photonics Rev.*.

[19] Engelberg J., Levy U. (2022). Standardizing flat lens characterization. *Nat. Photonics*.

[20] Engelberg J., Levy U. (2022). Generalized metric for broadband flat lens performance comparison. *Nanophotonics*.

[21] Yang F. (2021). Design of broadband and wide-field-of-view metalenses. *Opt. Lett.*.

[22] Luo S. (2022). Single-layer metalens for achromatic focusing with wide field of view in the visible range. *J. Phys. D Appl. Phys.*.

[23] Jang J., Lee G. Y., Kim Y., Kim C., Jeong Y., Lee B. (2023). Dispersion-Engineered metasurface doublet design for broadband and wide-angle operation in the visible range. *IEEE Photonics J.*.

[24] Hongli Y., Zhaofeng C., Xiaotong L. (2024). Broadband achromatic and wide field of view metalens-doublet by inverse design. *Opt. Express*.

[25] Levi L. (1980). *Applied Optics Vol. 2*.

[26] Goodman J. W. (1996). *Introduction to Fourier Optics*.

[27] Smith W. J. (2000). *Modern Optical Engineering*.

[28] Li Z. (2021). Meta-optics achieves RGB-achromatic focusing for virtual reality. *Sci. Adv.*.

[29] Decker M. (2019). Imaging performance of polarization-insensitive metalenses. *ACS Photonics*.

