# Supplementary material for: Nature inspired design methodology for a wide field of view achromatic metalens
Source: Nanophotonics. 2025 Oct 13;14(25):4531–43. doi: 10.1515/nanoph-2025-0279 (PMC12714054; doi:10.1515/nanoph-2025-0279)
Supplement: Supplementary file 1 — Supplementary Material Details [file j_nanoph-2025-0279_suppl_001.docx]

Supporting information

Nature Inspired Design Methodology for a Wide Field of View Achromatic Metalens

J. Engelberg,^1,2^ R. Mazurski,^1^, and U. Levy^1,^*

^1^Department of Applied Physics, The Faculty of Science, The Hebrew University of Jerusalem, Jerusalem, Israel, 9190401

^2^Department of Electro-optics and Applied Physics, Jerusalem College of Technology, Jerusalem, Israel, 9116001

[*ulevy@mail.huji.ac.il](mailto:*ulevy@mail.huji.ac.il)

# Resolution of human eye as a function of field

The resolution of the human eye on-axis (at the center of the fovea) is approximately 1 arcmin. This resolution degrades rapidly as the distance from the axis increases (this distance in terms of angular field-of-view is called the retinal eccentricity), as shown in Figure S1. The limiting factor for the resolution off-axis is the retina, but the optical resolution also degrades significantly. Figure S2 shows the optical MTF of the eye for fields-of-view from 0 to 30˚, based on an Eye model presented in MIL-HDBK-141 [1] and provided as Eyemodel2.len in the Code V optical design software sample lens library [2].


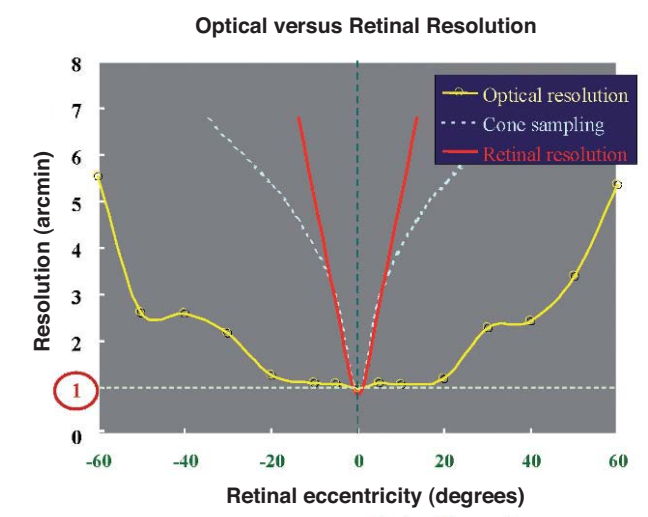


Figure S1: Optical resolution compared to cone sampling and retinal resolution. There is a good match at the fovea, but retinal resolution worsens much more rapidly with eccentricity than optical resolution (reproduced from  [3]).


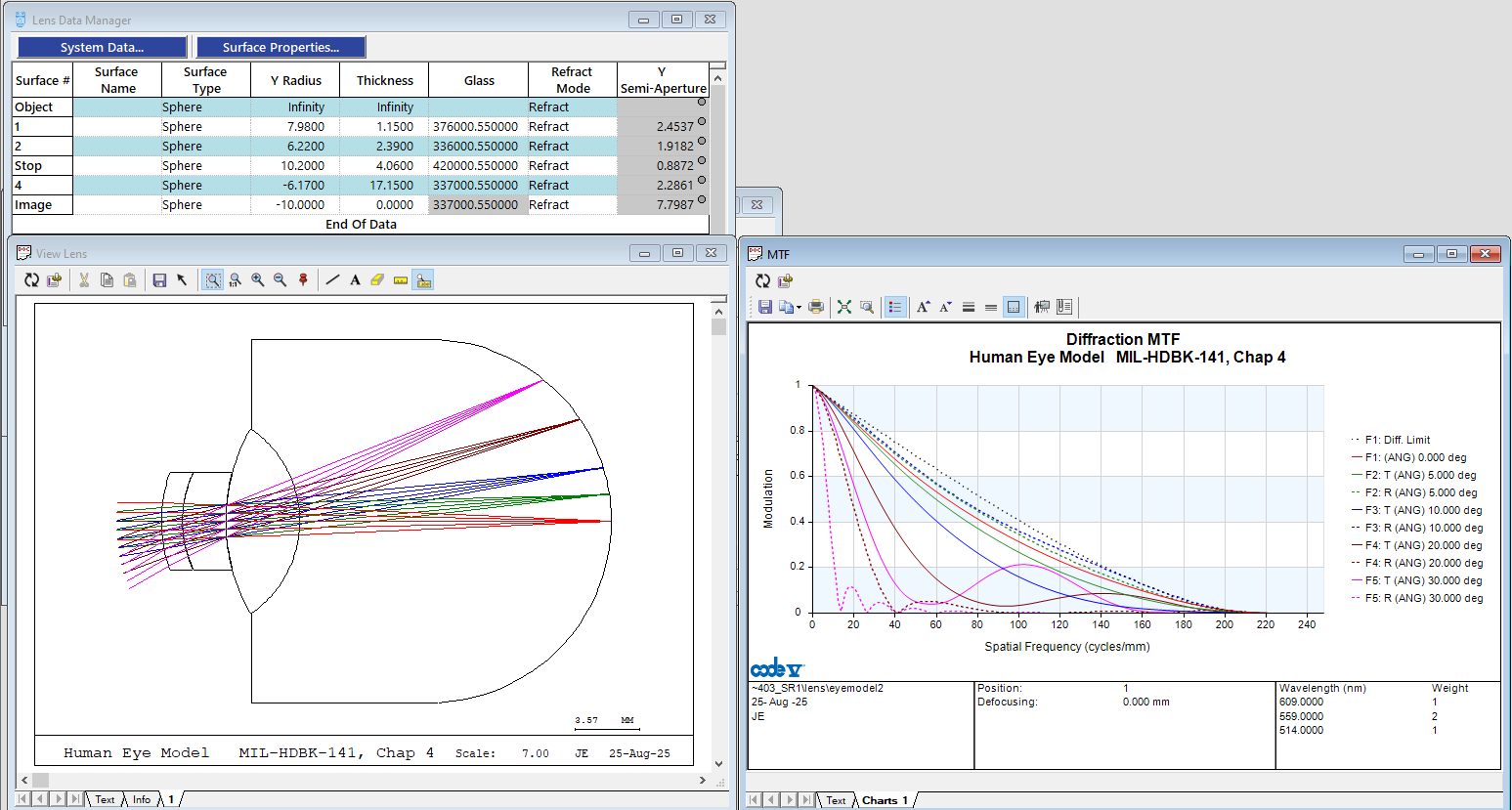


Figure S2: Lens data, optical layout and MTFs from Code V for eyemodel of MIL-HDBK-141, with 2mm pupil diameter. On-axis the MTF is near diffraction limited, but as the field angle increases the MTF drops.

# Zemax metalens prescriptions


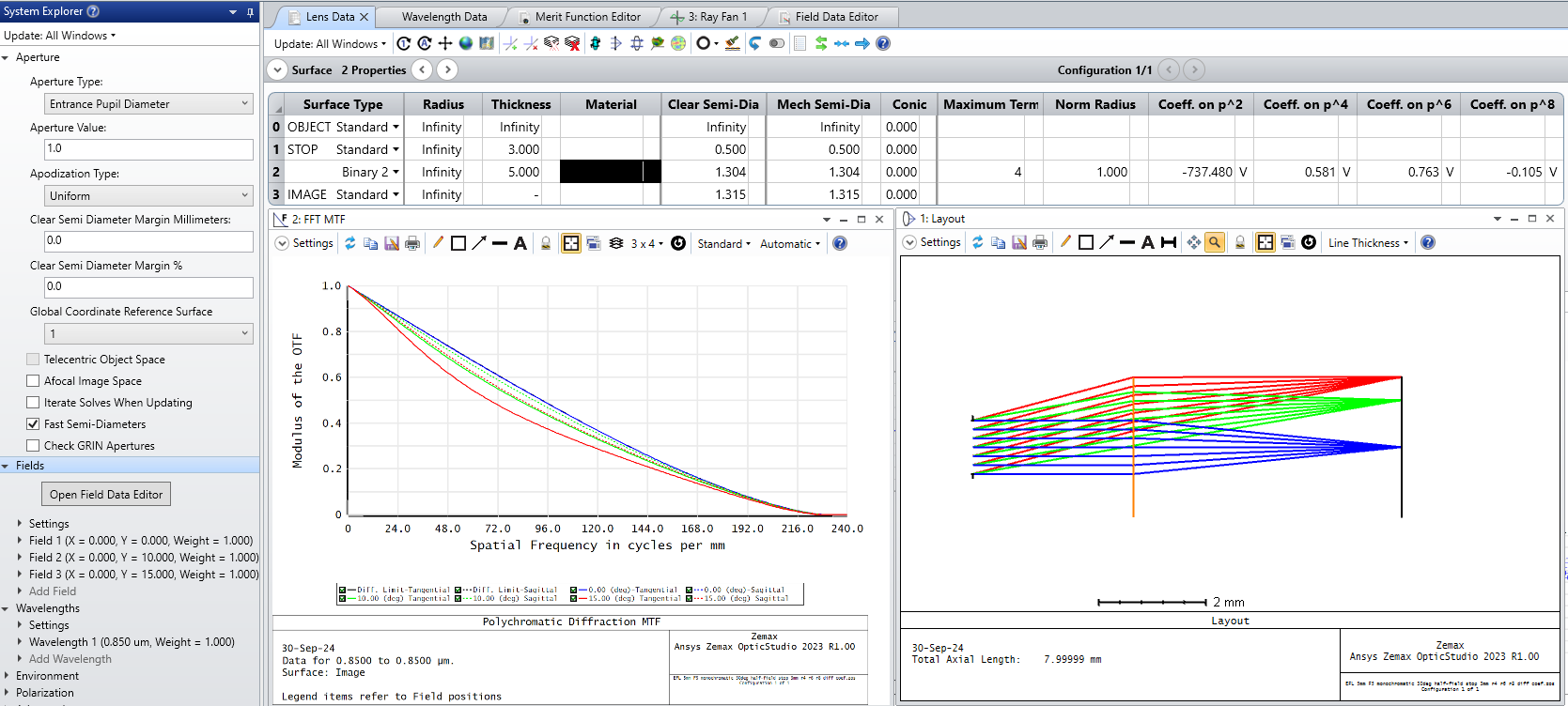


Figure S3: Metalens without substrate. Prescription and MTF performance, at 850nm single wavelength, 3mm stop distance, with high-order phase coefficients.


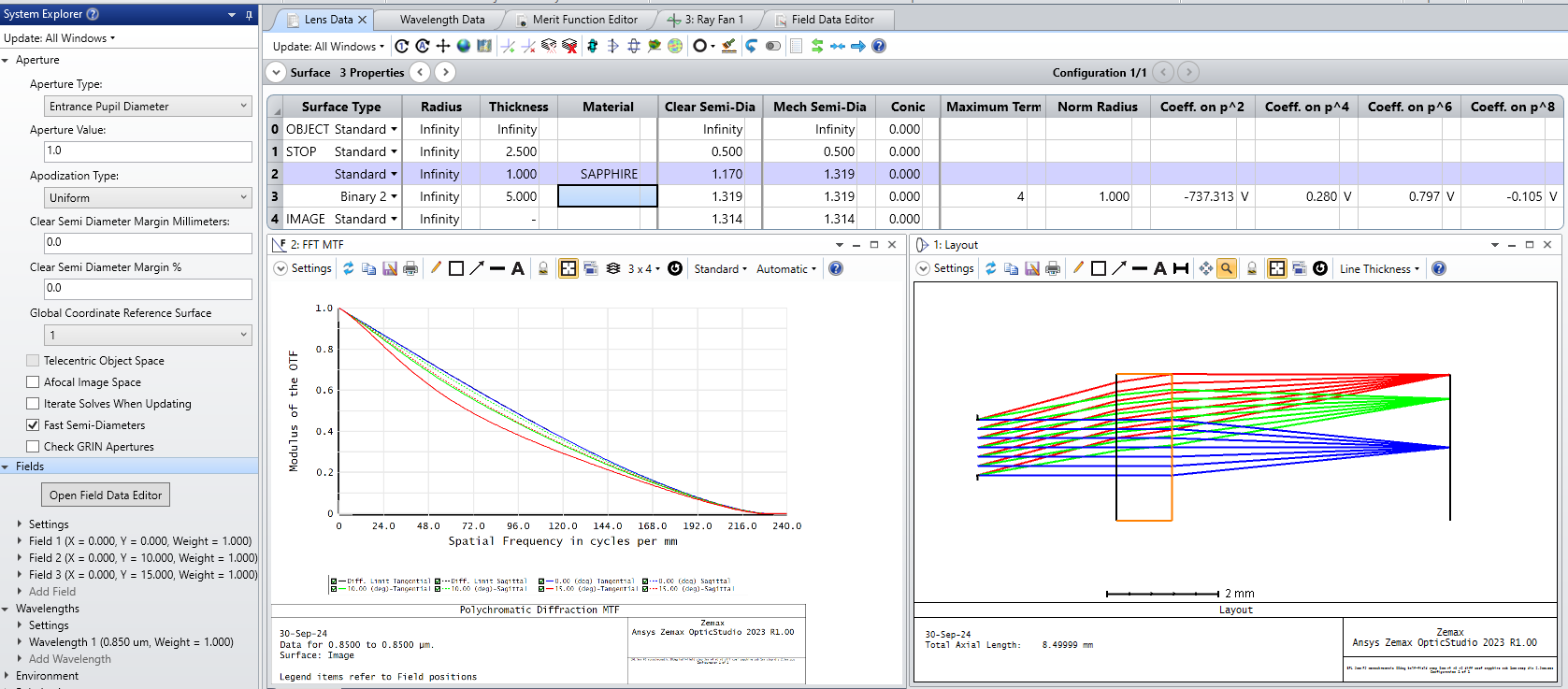


Figure S4: Metalens with 1mm thick sapphire substrate. Prescription and MTF performance, at 850nm single wavelength, 2.5mm stop distance from substrate front surface (metalens is on rear surface), with high-order phase coefficients.

The optical thickness of the 1mm substrate is given by:

$$t_{opt}=\frac{n-1}{n}t_{phys}=\frac{1.76-1}{1.76}\cdot1=0.43mm$$

Therefore, the overall equivalent stop distance for the design is 2.5+0.43=2.93mm. This is close to the 3mm design without substrate, which is why the two designs of Figure S33 and S4 give similar performance.

# Impact of phase jump

In our design we introduced a phase jump at the edge of the on-axis aperture, to remove the large off-axis aperture constraint which would limit the ability to correct the chromatic aberration on-axis. In this section we would like to refer to two questions:

1. What is the improvement in the on-axis performance obtained by introducing the phase jump?
2. What is the price paid in the off-axis performance to achieve this on-axis improvement?

To answer these questions, we use Figure S5 and Table S1. Let us begin with the first question. In Figure S5 we show the performance on-axis and 15˚off-axis. We can see that with the phase jump we obtain an on-axis OPM of 0.33, while without the phase-jump we obtain only 0.23 (see also Table S1). So, the improvement compared to the chromatic metalens on-axis OPM of 0.17 (see Table S1) is much more significant with the phase jump. The improvement in OPM is due to an improvement in both diffraction efficiency and Strehl ratio.

To answer the second question, we compare the achromatic metalens performance at 15˚ off-axis with and without the phase jump. Off-axis the phase jump improves efficiency slightly but does not improve the Strehl ratio significantly and in some cases even degrades it (see Table S1). This is not surprising, since a phase jump in the aperture is expected to create a wavefront error that degrades the MTF. The two effects tend to cancel each other, and in the end the off-axis OPM is still slightly better in all cases with the phase jump than without the phase jump. We do not expect the phase jump to affect tolerances or produce imaging artifacts.


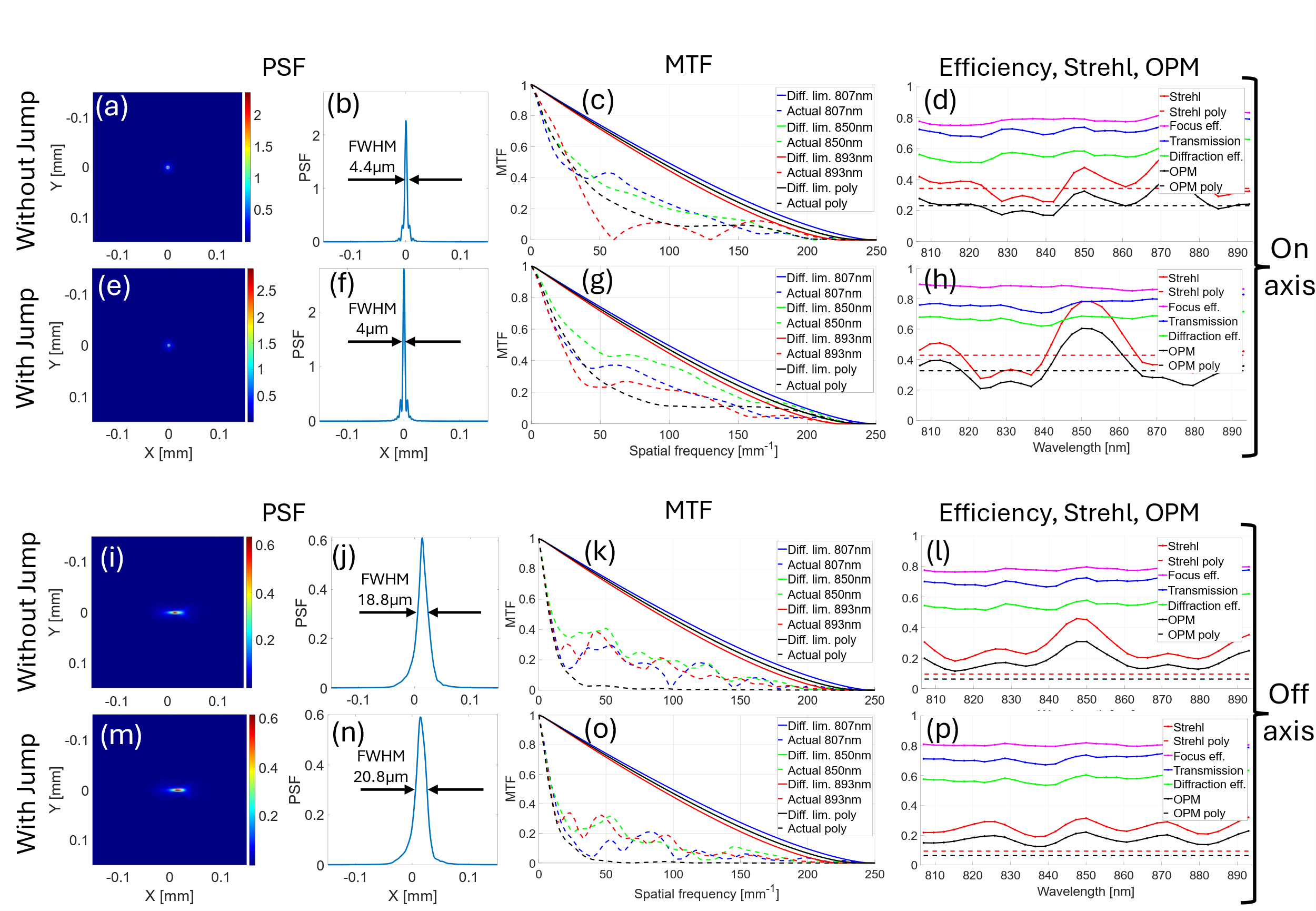


Figure S5: On-axis and 15˚ off-axis performance comparison between achromatic metalens with (e-h, m-p) and without (a-d, i-l) a phase jump. (a, e, i, m) 2D PSFs normalized so that the volume under the function is equal to 1, and (b, f, j, n) their cross sections, respectively. FWHM is marked on each PSF’s cross-section. (c, g, k, o) MTF comparison, solid lines represent diffraction limited MTFs, and dashed lines are the achieved MTFs. The dashed black lines are the polychromatic MTFs. (d, h, l, p) Strehl, efficiency and overall performance (OPM) comparison as a function of wavelength. Evaluation is at the optimal stop distance of 2.5mm.

| 20⁰ | 15⁰ | 10⁰ | 5⁰ | 0⁰ | Field Angle | |
| --- | --- | --- | --- | --- | --- | --- |
| 53.2 | 28.4 | 13.2 | 6.4 | 4.4 | Chromatic | Poly. FWHM $[\mu m]$ |
| 27.6 | 18.8 | 12.0 | 7.2 | 4.4 | Achromatic No phase jump |  |
| 28.8 | 20.8 | 12.0 | 7.6 | 4.0 | Achromatic with phase jump |  |
| 0.046 | 0.071 | 0.124 | 0.221 | 0.302 | Chromatic | Poly. Strehl |
| 0.063 | 0.095 | 0.144 | 0.232 | 0.343 | Achromatic No phase jump |  |
| 0.061 | 0.094 | 0.150 | 0.232 | 0.428 | Achromatic with phase jump |  |
| 0.457 | 0.462 | 0.470 | 0.472 | 0.473 | Chromatic | Poly. Diffraction Efficiency |
| 0.555 | 0.551 | 0.572 | 0.577 | 0.573 | Achromatic No phase jump |  |
| 0.569 | 0.576 | 0.602 | 0.609 | 0.666 | Achromatic with phase jump |  |
| 0.025 | 0.040 | 0.070 | 0.125 | 0.170 | Chromatic | Poly. OPM |
| 0.041 | 0.062 | 0.097 | 0.158 | 0.231 | Achromatic No phase jump |  |
|  |  |  |  |  |  |  |
| 0.041 | 0.064 | 0.106 | 0.165 | 0.326 | Achromatic with phase jump |  |

Table S1: Performance comparison between chromatic metalens, and achromatic metalens with and without a phase jump.

To gain a better understanding of the effect of the phase jump on the Strehl ratio, we simulated the MTF for the case of an ideal phase profile (i.e., the target phase used when assigning our nanostructures), thus eliminating the effects of chromatic aberration. We then looked at the MTF for the ideal phase profiles with and without the phase jump, for each of the field points, for a stop distance of 2.5mm (the distance used for the achromatic metalens). It can be seen in Figure S6 (a) that on-axis the phase jump has no effect, which makes sense, since the jump occurs only at the edge of the on-axis aperture. As we move to the intermediate off-axis angles of 5˚ and 10˚ the MTF degrades significantly because of the phase jump. However, as we move to larger off-axis angles the degradation is less significant, for two reasons: (a) The MTF with no phase jump is no longer diffraction limited, because of the reduced stop distance, which introduces coma aberration. (b) The phase jump is located closer to the edge of the aperture so the external area where the phase is shifted is more negligible. We added the 25˚ field point to demonstrate that at this angle there is again no degradation, since the aperture is entirely beyond the phase jump radius of 0.5mm.

Comparing the MTFs of Figure S6 to the overall MTFs shown in Figures S8 (a), S10 (a), 4 (k), and S12 (a) (which include the effects chromatic aberrations), we can see that the overall MTF is significantly worse than the MTFs of Figure S6. This explains why the degradation in Strehl ratio for the off-axis points is small if any.


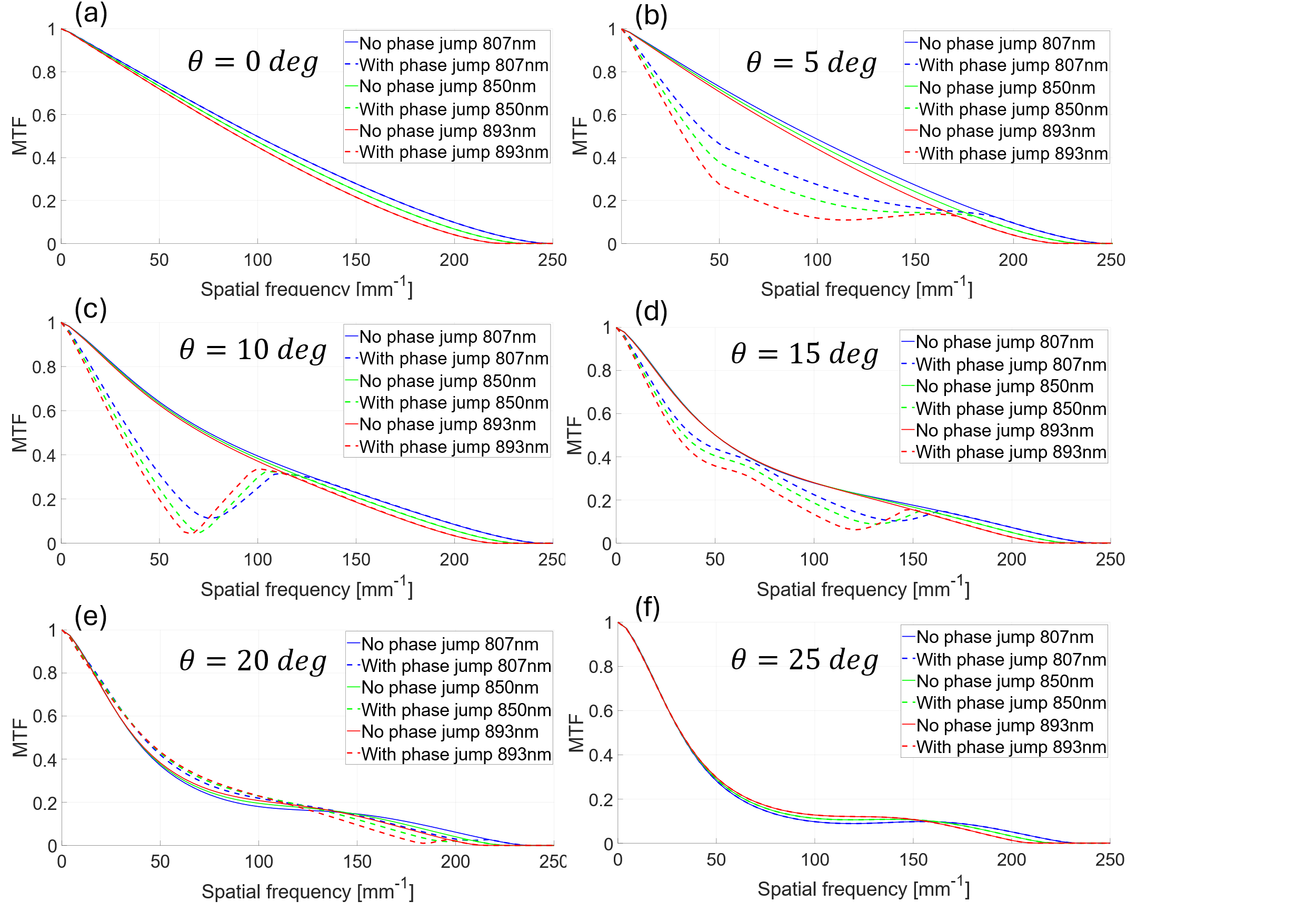


Figure S6: MTF degradation because of phase jump. (a) On-axis (b) 5˚ off-axis (c) 10˚ off-axis (d) 15˚ off-axis (e) 20˚ off-axis (f) 25˚ off-axis.

# Achromatic metalens polychromatic performance at additional off-axis points


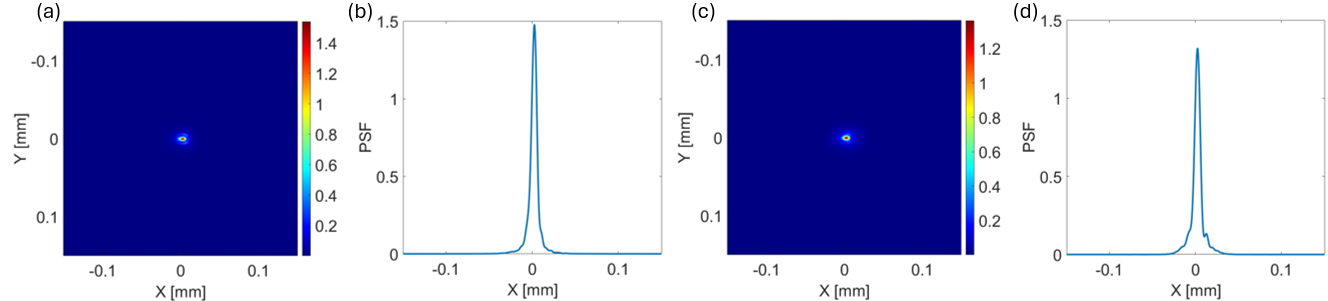


Figure S7: 5˚ off-axis PSF comparison at stop distance of 2.5mm. (a-b) Achromatic metalens 2D PSF and cross-section in the radial (horizontal) direction respectively. (c-d) Chromatic metalens 2D PSF and cross-section in the radial (horizontal) direction respectively.

###
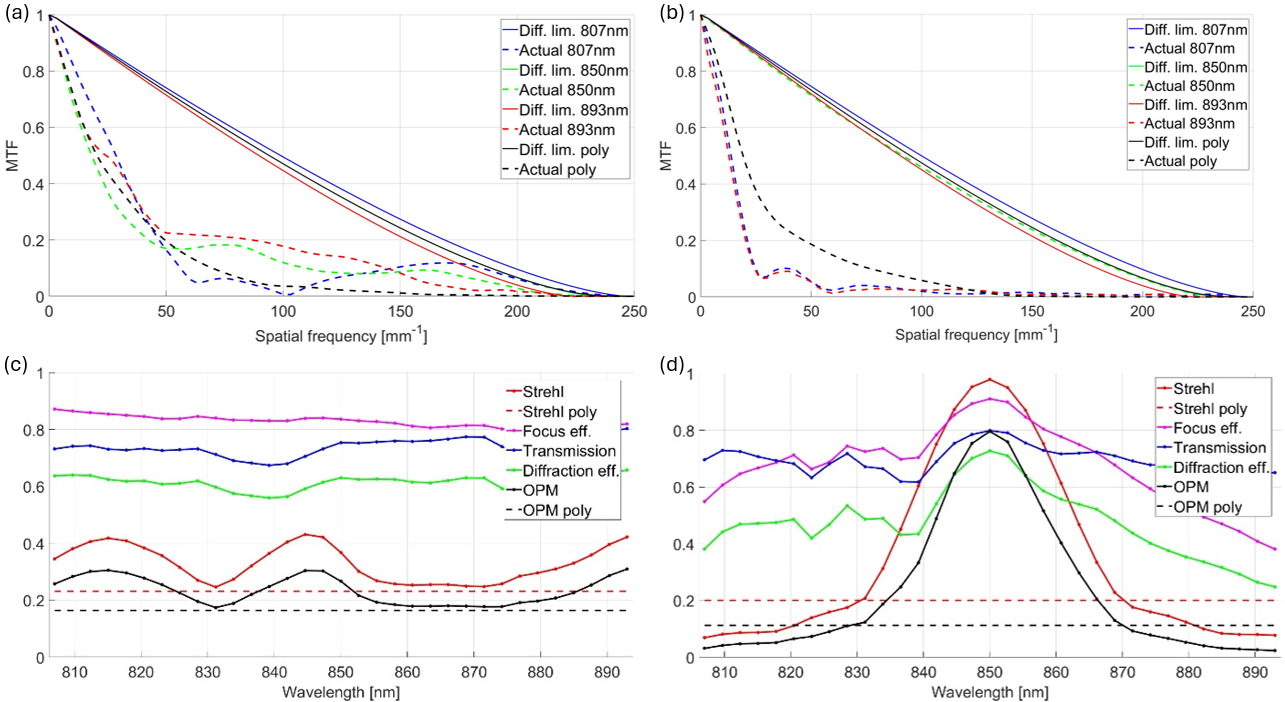


Figure S8: 5˚ off-axis performance comparison at stop distance of 2.5mm. (a) Achromatic metalens MTF. (b) Chromatic metalens MTF. (c) Achromatic metalens spectral and polychromatic Strehl, efficiency and overall performance (OPM) results as a function of wavelength. (d) The same for chromatic metalens.

###


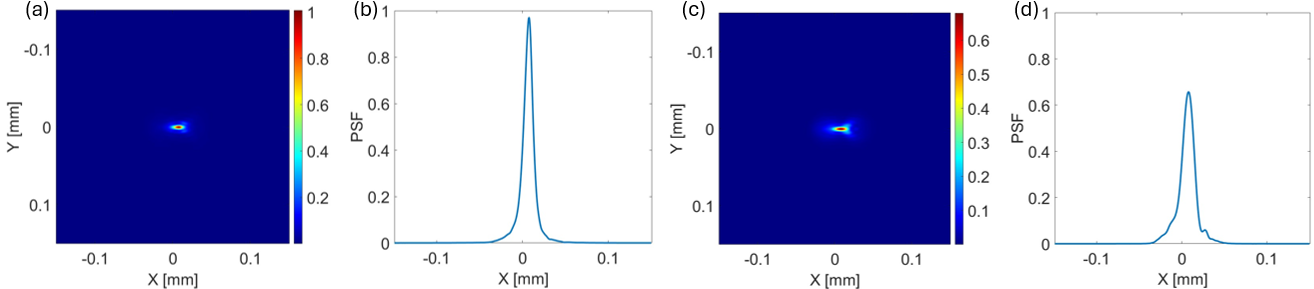


Figure S9: 10˚ off-axis PSF comparison at stop distance of 2.5mm. (a-b) Achromatic metalens 2D PSF and cross-section in the radial (horizontal) direction respectively. (c-d) Chromatic metalens 2D PSF and cross-section in the radial (horizontal) direction respectively.


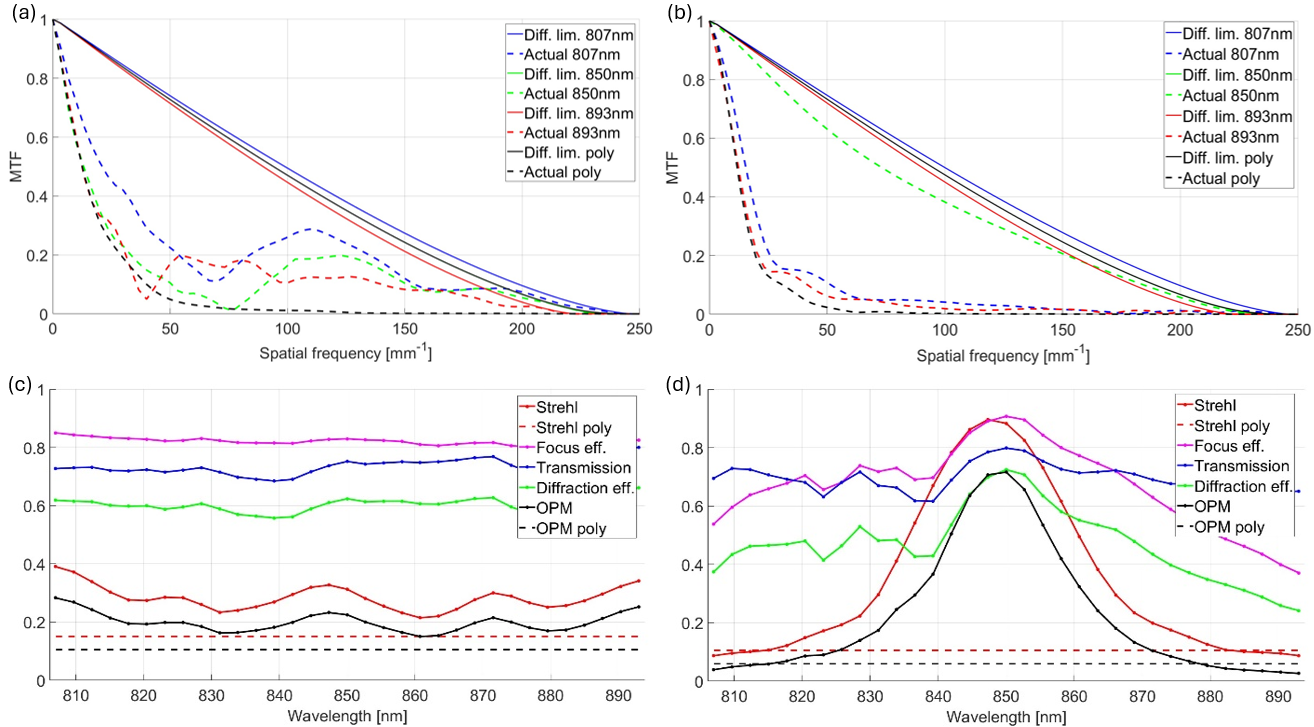


Figure S10: 10˚ off-axis performance comparison at stop distance of 2.5mm. (a) Achromatic metalens MTF. (b) Chromatic metalens MTF. (c) Achromatic metalens spectral and polychromatic Strehl, efficiency and overall performance (OPM) results as a function of wavelength. (d) The same for chromatic metalens.


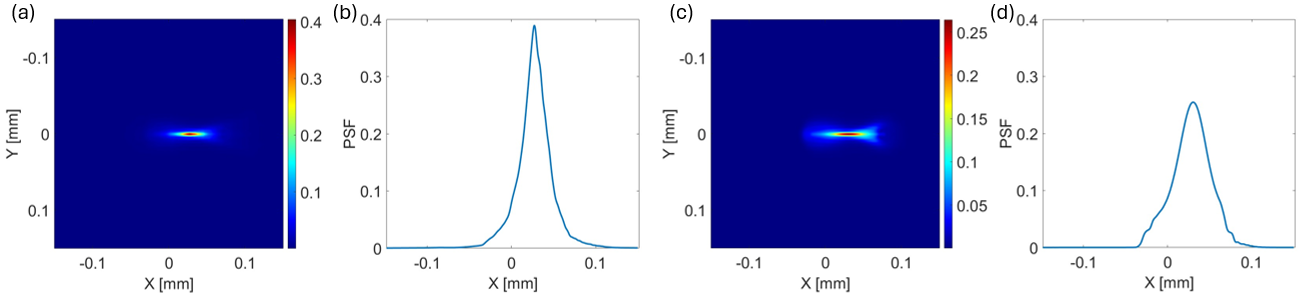


Figure S11: 20˚ off-axis PSF comparison at stop distance of 2.5mm. (a-b) Achromatic metalens 2D PSF and cross-section in the radial (horizontal) direction respectively. (c-d) Chromatic metalens 2D PSF and cross-section in the radial (horizontal) direction respectively.


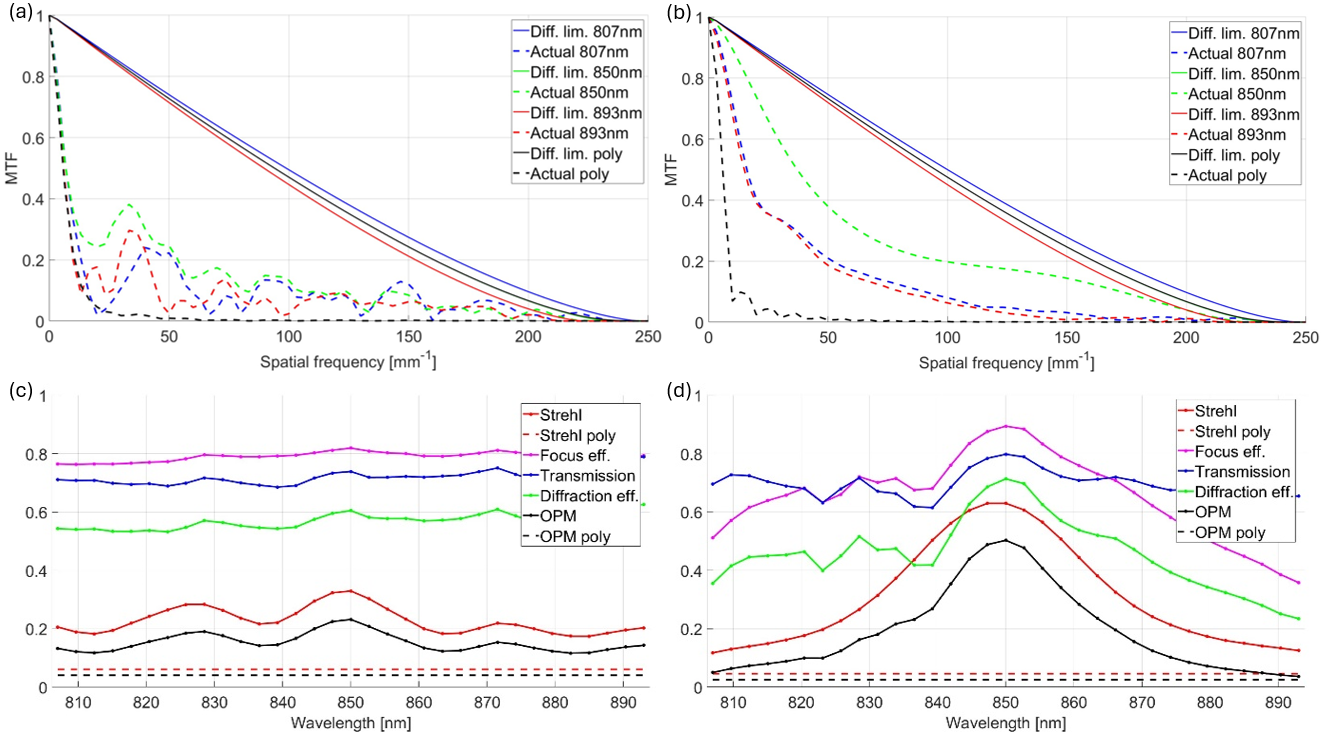


Figure S12: 20˚ off-axis performance comparison at stop distance of 2.5mm. (a) Achromatic metalens MTF. (b) Chromatic metalens MTF. (c) Achromatic metalens spectral and polychromatic Strehl, efficiency and overall performance (OPM) results as a function of wavelength. (d) The same for chromatic metalens.

The center of each PSF image, which is marked as $x=y=0$, is located at a radial (horizontal in our images) distance of $fsin(\theta)$ relative to the optical axis. This is the chief ray height for the case of a quadratic phase metalens with stop at the front focal plane [4]. For this reason, when the aperture is not at the front focal plane and/or the phase is not quadratic, one can see that the center of the off-axis spot is not exactly on the center of the image. This does not affect the MTF results.

# Achromatic metalens single wavelength performance

Following are images of PSFs at 3 single wavelengths (S13 – S17). It can be seen that the lateral chromatic aberration is partially corrected by the achromatic metalens by looking at off-axis PSFs (S14-S17): For the achromatic metalens the PSFs are approximately centered on the same *x* position (for each field angle) although they may seem distorted. Conversely, the chromatic metalens PSFs are shifted to the right for the short wavelength (807nm) and to the left for the long wavelength (893nm).

Another insight from the individual wavelengths PSFs is the ability to correct the axial chromatic aberration, as can be seen by looking at the peaks of the PSFs that are all on the same order of magnitude for the achromatic metalens, meaning similar Strehl ratio for all wavelengths. Conversely, for the chromatic metalens it is obvious that the enormous difference between the peaks of the design wavelength and the edge wavelengths is caused by defocusing of the latter (note that the chromatic metalens PSF Y-axis is different for the center wavelength and the extreme wavelengths).


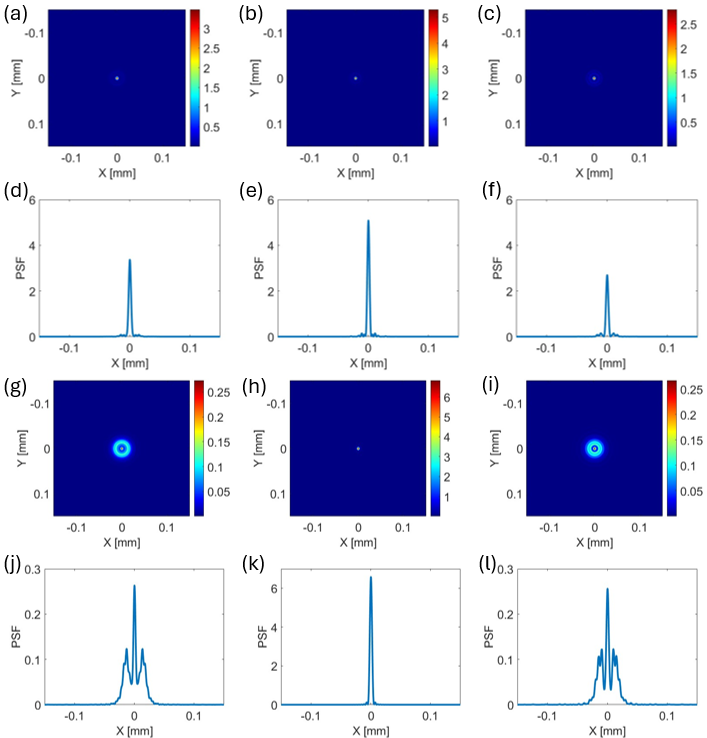


Figure S13: On-axis PSF comparison for individual wavelengths. (a-f) Achromatic metalens. (g-l) Chromatic metalens. (a) and (g) are the PSFs at 807nm for achromatic and chromatic metalens, respectively. (d) and (j) are their cross sections, respectively. (b) and (h) are the PSFs at 850nm for achromatic and chromatic metalens, respectively. (e) and (k) are their cross sections, respectively. (c) and (i) are the PSFs at 893nm for achromatic and chromatic metalens, respectively. (f) and (l) are their cross sections, respectively.


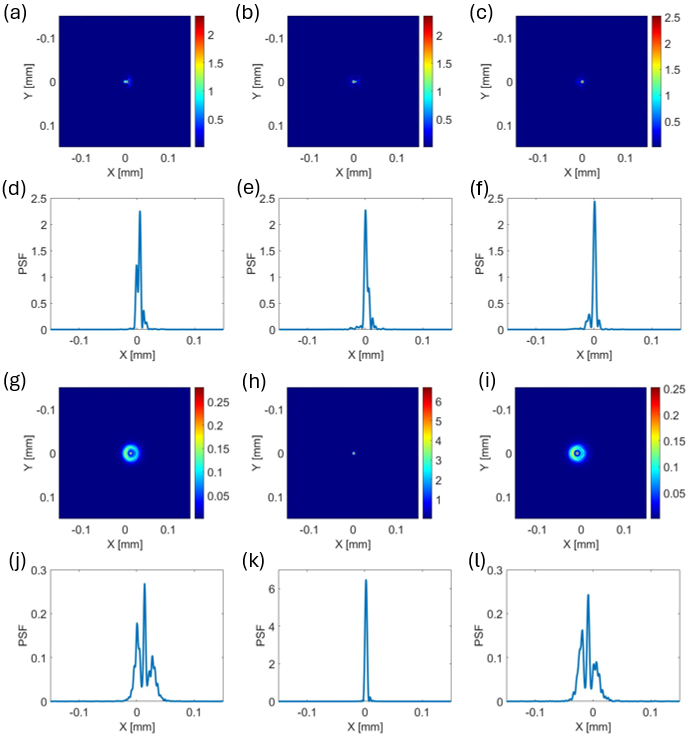


Figure S14: 5˚ off-axis PSF comparison for individual wavelengths. (a-f) Achromatic metalens. (g-l) Chromatic metalens. (a) and (g) are the PSFs at 807nm for achromatic and chromatic metalens, respectively. (d) and (j) are their cross sections, respectively. (b) and (h) are the PSFs at 850nm for achromatic and chromatic metalens, respectively. (e) and (k) are their cross sections, respectively. (c) and (i) are the PSFs at 893nm for achromatic and chromatic metalens, respectively. (f) and (l) are their cross sections, respectively.


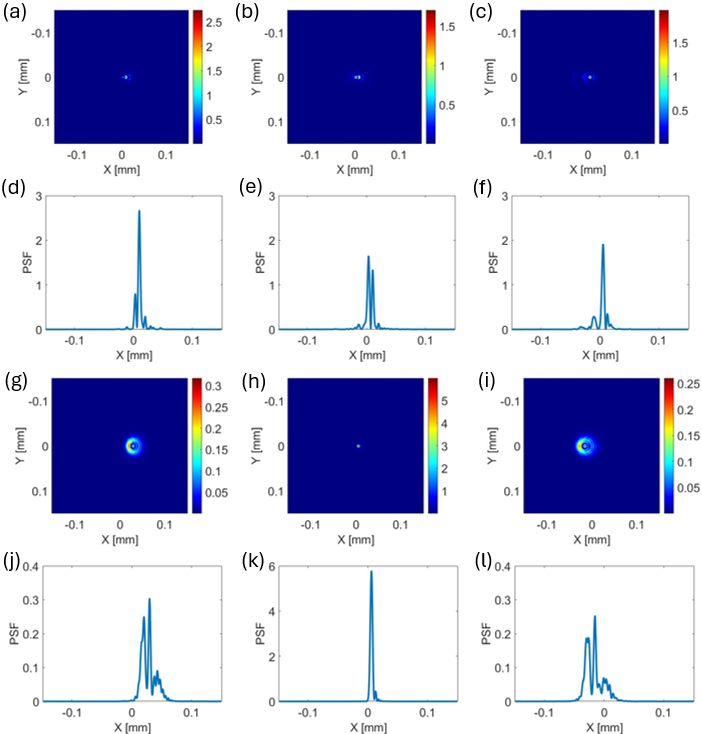


Figure S15: 10˚ off-axis PSF comparison for individual wavelengths. (a-f) Achromatic metalens. (g-l) Chromatic metalens. (a) and (g) are the PSFs at 807nm for achromatic and chromatic metalens, respectively. (d) and (j) are their cross sections, respectively. (b) and (h) are the PSFs at 850nm for achromatic and chromatic metalens, respectively. (e) and (k) are their cross sections, respectively. (c) and (i) are the PSFs at 893nm for achromatic and chromatic metalens, respectively. (f) and (l) are their cross sections, respectively.


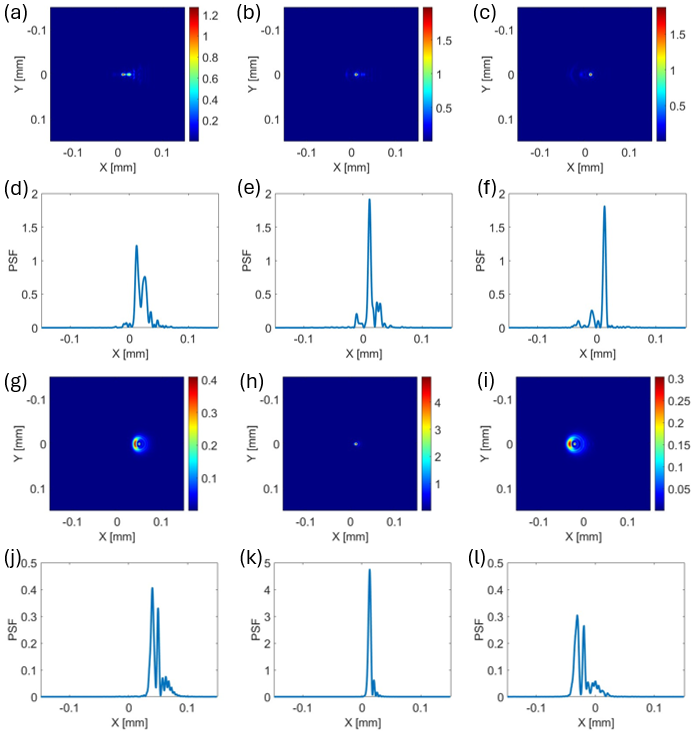


Figure S16: 15˚ off-axis PSF comparison for individual wavelengths. (a-f) Achromatic metalens. (g-l) Chromatic metalens. (a) and (g) are the PSFs at 807nm for achromatic and chromatic metalens, respectively. (d) and (j) are their cross sections, respectively. (b) and (h) are the PSFs at 850nm for achromatic and chromatic metalens, respectively. (e) and (k) are their cross sections, respectively. (c) and (i) are the PSFs at 893nm for achromatic and chromatic metalens, respectively. (f) and (l) are their cross sections, respectively.


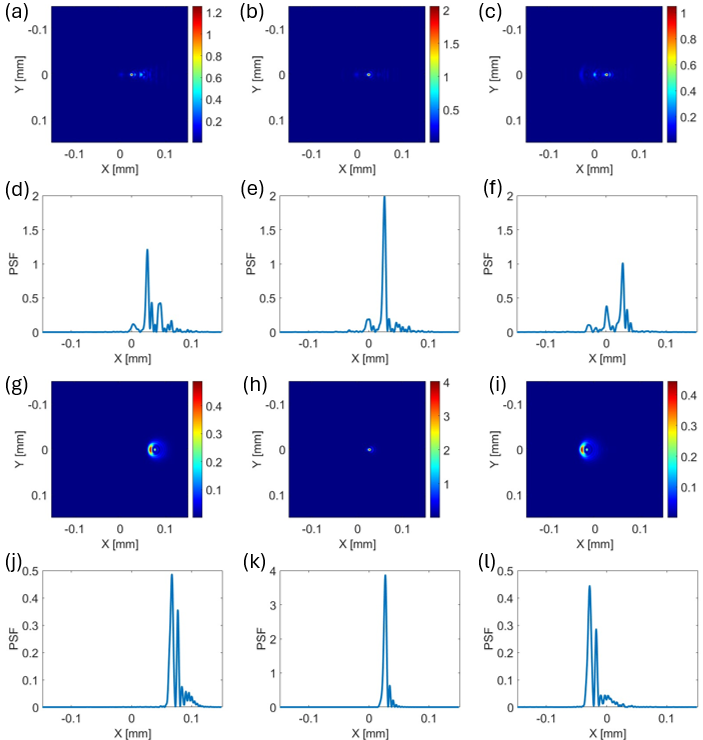
Figure S17: 20˚ off-axis PSF comparison for individual wavelengths. (a-f) Achromatic metalens. (g-l) Chromatic metalens. (a) and (g) are the PSFs at 807nm for achromatic and chromatic metalens, respectively. (d) and (j) are their cross sections, respectively. (b) and (h) are the PSFs at 850nm for achromatic and chromatic metalens, respectively. (e) and (k) are their cross sections, respectively. (c) and (i) are the PSFs at 893nm for achromatic and chromatic metalens, respectively. (f) and (l) are their cross sections, respectively.

# References

1. *Military Standardization Handbook - Optical Design, MIL-HDBK-141* (Department of Defense, USA, 1962).

2. *Code V Lens System Setup Reference Manual* (Synopsys, 2024).

3. R. Navarro, "The optical design of the human eye: A critical review," J Optom **2**, 3–18 (2009).

4. D. A. Buralli and G. M. Morris, "Design of a wide field diffractive landscape lens," Applied Optics **28**, 3950–3959 (1989).
